# Maintenance of caecal homeostasis by diverse adaptive immune cells in the rhesus macaque

**DOI:** 10.1002/cti2.1508

**Published:** 2024-05-02

**Authors:** Xaquin Castro Dopico, Mariia Guryleva, Marco Mandolesi, Martin Corcoran, Jonathan M Coquet, Ben Murrell, Gunilla B Karlsson Hedestam

**Affiliations:** ^1^ Department of Microbiology, Tumor and Cell Biology Karolinska Institutet Stockholm Sweden; ^2^ Department of Immunology and Microbiology University of Copenhagen Copenhagen DK Denmark

**Keywords:** caecum, gut homeostasis, rhesus macaque, single‐cell RNA sequencing, tissue atlas

## Abstract

**Objectives:**

The caecum bridges the small and large intestine and plays a front‐line role in discriminating gastrointestinal antigens. Although dysregulated in acute and chronic conditions, the tissue is often overlooked immunologically.

**Methods:**

To address this issue, we applied single‐cell transcriptomic‐V(D)J sequencing to FACS‐isolated CD45^+^ caecal patch/lamina propria leukocytes from a healthy (5‐year‐old) female rhesus macaque *ex vivo* and coupled these data to VDJ deep sequencing reads from haematopoietic tissues.

**Results:**

We found caecal NK cells and ILC3s to co‐exist with a spectrum of effector T cells partially derived from *SOX4* ^+^ recent thymic emigrants. Tolerogenic Vγ8Vδ1‐T cells, plastic CD4^+^ T helper cells and *GZMK* ^+^ *EOMES* ^+^ and *TMIGD2* ^+^ tissue‐resident memory CD8^+^ T cells were present and differed metabolically. An *IL13* ^+^ *GATA3* ^+^ Th_2_ subset expressing eicosanoid pathway enzymes was accompanied by *IL1RL1* ^+^ *GATA3* ^+^ regulatory T cells and a minor proportion of IgE^+^ plasma cells (PCs), illustrating tightly regulated type 2 immunity devoid of ILC2s. In terms of B lymphocyte lineages, caecal patch antigen‐presenting memory B cells sat alongside germinal centre cells undergoing somatic hypermutation and differentiation into *IGF1* ^+^ PCs. Prototypic gene expression signatures decreased across PC clusters, and notably, expanded IgA clonotypes could be traced in VDJ deep sequencing reads from additional compartments, including the bone marrow, supporting that these cells contribute a steady stream of systemic antibodies.

**Conclusions:**

The data advance our understanding of caecal immunological function, revealing processes involved in barrier maintenance and molecular networks relevant to disease.

## Graphical abstract

The caecal mucosa plays a front‐line role discriminating pathogens from commensals at the start of the large intestine, yet how the tissue contributes to acute and inflammatory gastrointestinal conditions is poorly understood, especially in primates. To this end, we used top‐down single‐cell sequencing to illustrate a diverse network of T and B lymphocytes that patrol barrier in the steady state, pin‐pointing distinct tolerogenic and inflammatory cell types, and immunological mechanisms of clinical relevance.

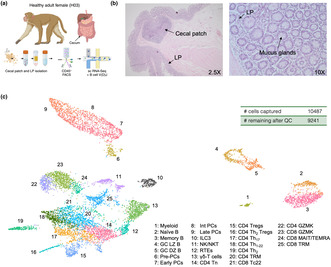

## Introduction

Rhesus macaques (*Macaca mulatta*) are a major success story of the Anthropocene and an important model organism for understanding host–pathogen interactions and evaluating medical interventions.^1^ This makes characterisation of immunological compartments in this species essential for effective translational and veterinary medicine.

The gastrointestinal tract harbours an abundance of effector‐memory lymphocytes maintaining commensal tolerance and preventing pathogenic infection that impinge upon intestinal pathology and organism‐wide physiology.^2^ In recent years, this compartment has been increasingly mapped in humans and laboratory mice using single‐cell RNA sequencing, yielding several new insights.^3^, ^4^ At present, high‐resolution genomic studies of caecal and vermiform appendix lymphoid cells in primates are absent from the literature.

Linking the small and large intestines, the caecal pouch (present in most amniotes) hosts a population of microbes that furthers the breakdown of dietary matter before it enters the colon.^5^ As O_2_ tension decreases from the proximal small intestine to the caecum and foreign organism biomass increases by orders of magnitude, lymphocytes in the caecum play an important role discriminating opportunistic pathogens from commensals.^6^, ^7^ In rhesus macaques, the caecum forms a distinctive pouch ending at the apex caeci. Interestingly, in some old‐world monkeys (but not rhesus macaques) and humans, the caecum extends into the finger‐like vermiform appendix,^8^ posited to be a ‘safe‐house’ for symbiotic bacteria.^9^ Anatomically it seems plausible that responses engendered in the caecum may influence phenotypes more distally in the gastrointestinal tract, consistent with murine caecal plasma cells (PCs) disseminating throughout the small and large intestines, while Peyer's patch PCs were restricted to the small bowel.^10^

Like typhlitis (neutropenic enterocolitis), appendicitis can prove fatal, although the triggers for and molecular dysregulations underpinning these conditions are largely unknown, with most human studies considering the caecum analogous to the ascending colon, despite its distinctive anatomy.^11^ Various conditions, such as bacterial and viral infections, can lead to an oedematous caecal mucosa and increased lamina propria (LP) cell numbers,^12^ although such observations are rarely made in practice. It is notable that environmental enteric dysfunction in rhesus macaques is associated with histopathological changes along the large intestine (including the caecum) and reduced infant growth rates,^13^ and that adenocarcinoma of the ileocecocolic junction, caecum or colon is the most common spontaneous neoplasm in captive animals.^14^

In humans, ‘caecal patch lesions’ are manifestations of ulcerative colitis (UC) that can aid diagnosis.^15^ In such cases, inflammation is present around the appendiceal orifice and has a similar endoscopic appearance to lesions in the rectum. In macaques, cicatrising UC of the caecum has been described,^16^ indicating caecal–appendiceal involvement in inflammatory bowel disease in some individuals.

In the acute context, neutropenic enterocolitis is primarily caused by enteric infections and is more common in immunocompromised individuals, such as in HIV/AIDS and cancer patients, and the elderly.^17^, ^18^ In severe cases, dysregulated host inflammation contributes to a loss of barrier integrity and bacteraemia.^19^ Indeed, the caecal mucosa is rapidly invaded by intestinal bacteria post‐mortem,^20^ highlighting the high energetic demand of homeostasis. To maintain the barrier, caecal patches (CPs) – not caecal patch lesions – are distributed across the mucosa. Prior immunophenotyping in this species has reported that the caecal mucosa is characterised by an abundance of effector‐memory T cells and IgA‐/IgM‐secreting PCs,^21^, ^22^ with flow cytometric studies indicating a close resemblance between macaque and human gastrointestinal immune cells.^23^, ^24^

We dissected the caecum post‐mortem from a healthy, 5‐year‐old female (H03) terminated at the conclusion of another study. By focusing on a single sample *ex vivo*, we hoped to capture endogenously activated pathways and cell states in high resolution. Given the importance of gut‐derived PCs for the mucosal barrier, we defined the antibody repertoire alongside the transcriptome of CD45^+^ leucocytes. We coupled the single‐cell data to immunoglobulin (IG) heavy (IGH)‐chain VDJ deep sequencing reads generated from additional compartments to test for caecal PC clonotype dissemination.

## Results

**Figure 1:**
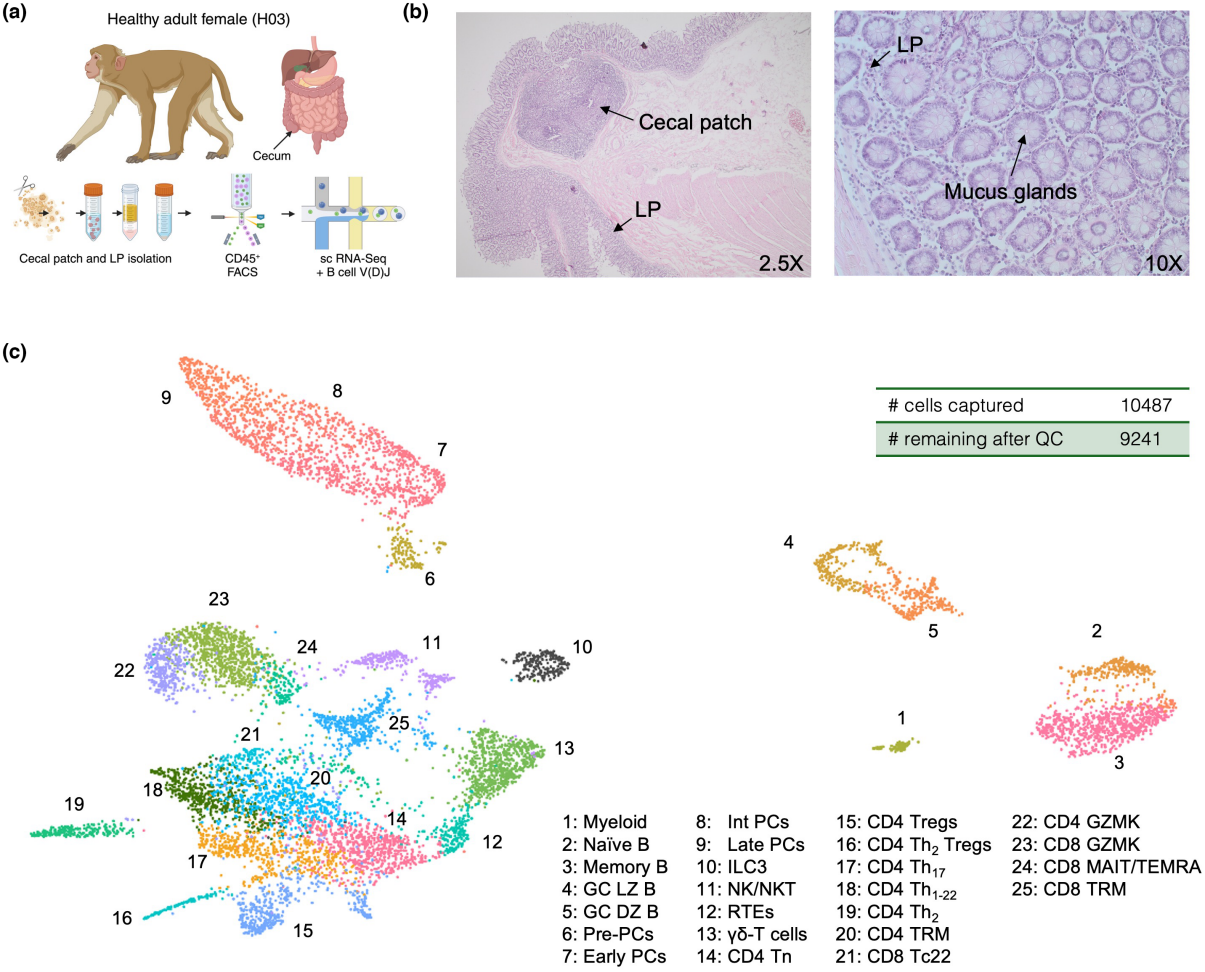
An adaptive immune cell atlas of the rhesus macaque caecum. **(a)** Study design: the caecum was dissected at termination from H03 and processed for the enrichment of LP and CP leucocytes. *Ex vivo* live CD45^+^ cells (200 000) isolated by FACS were diluted and loaded on to one channel of a 10× Chromium chip. **(b)** H&E staining of the caecum from H03. CP, LP and mucous glands are indicated by arrows. Magnification inset. **(c)** UMAP of whole dataset based on 3000 highly differentially expressed genes. A total of 10 487 cells were captured by the experiment: 9241 remained after QC (see the Methods). Cluster numbers and corresponding cell‐type annotations are inset.

The caecum was dissected post‐mortem, and the apex processed for the enrichment of CP and LP leucocytes. Live CD45^+^ cells from the lymphocyte gate were isolated by FACS and re‐suspended for single‐cell genomics (Figure 1a, Supplementary figure 1a, b). Histology showed CPs had light and dark zones (LZ and DZ), indicative of germinal centres (GCs), and were in close association with the LP and vasculature (Figure 1b). After QC, UMAP analysis of single‐cell data projected 25 clusters from 9241 cells (Figure 1c). Broadly, cells separated into T cells and innate lymphoid cells (ILCs) in one sector of the projection, and B cells and PCs in another, with proliferating GC B cells forming a distinctive population (Figure 1c).

The small cluster of myeloid cells (< 1%) differentially expressed *IL1B*, *AIF1*, *LYZ*, *IDO1* and *APOBEC3A*, and displayed up‐regulation of tryptophan and kynurenine metabolism genes^25^ (Supplementary figure 1c–e; Supplementary table 1).

**Figure 2:**
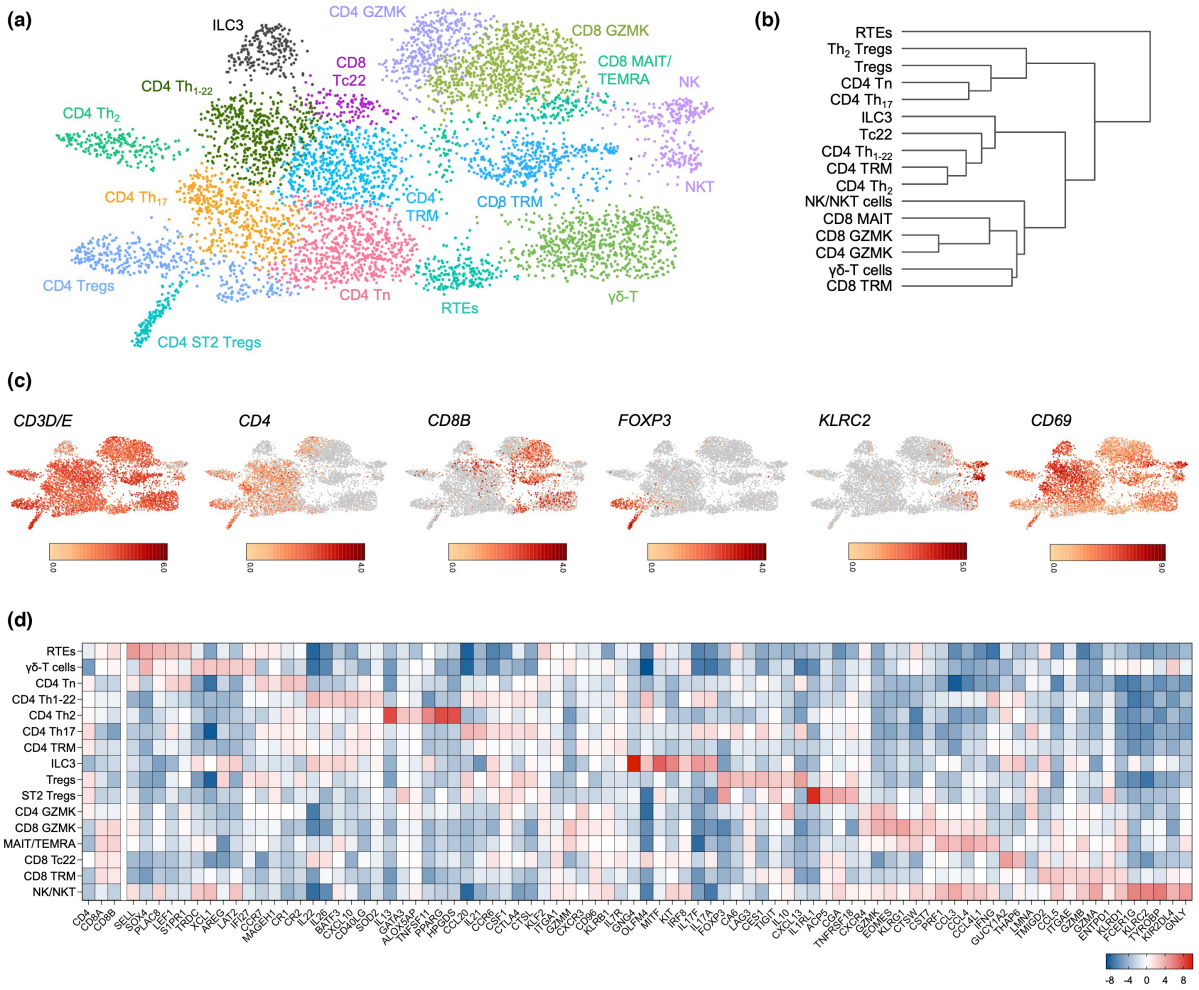
T‐cell and ILC populations of the rhesus macaque caecum. **(a)** UMAP projection of re‐clustered T cells and ILCs. Cell‐type annotations are inset**. (b)** Clustering of T‐cell and ILC populations based on 3000 highly DE genes. **(c)** Expression of key lineage marker genes among T cells and ILCs. Log_2_ expression for each marker is indicated by the scale bar. **(d)** Heatmap showing the most differentially expressed genes per cluster. Log_2_ fold‐change is shown.

To resolve remaining cells, ILCs and T cells, and B lineage cells were re‐clustered independently, excluding the myeloid cells (Supplementary figure 2). T cells and ILCs contributed 65% of the total dataset and were assigned to 16 clusters (Figure 2a and b, Supplementary figure 2). Approximately 50% were *CD4* ^+^ T cells, while one quarter corresponded to *CD8B* ^+^ T cells (Figure 2c). Approximately one third of γδ‐T cells (11% of total) also expressed *CD8B*. The majority of cells were T‐cell receptor alpha variable (*TRAV*)‐ and/or T‐cell receptor beta variable (*TRBV*)‐expressers. Expression of a T‐cell receptor delta constant (*TRDC* – *ENSMMUG00000057791*) amplicon was observed in ILCs and γδ‐T cells, as previously reported^26^ (Supplementary figure 3a). The majority of T cells and ILCs expressed the marker of tissue residency, *CD69* (74% > 1 copy), while other markers of T‐cell tissue residency were more heterogeneously expressed (Supplementary figure 3b). Strongly differentially expressed genes between T and ILC clusters are shown in Figure 2d, such as *IL1RL1* and *EOMES*, which delineated ST2^+^ Tregs and *GZMK* ^+^ T cells respectively.

### Cytotoxic NK cells and ILC3s form the core ILC populations

A *TRDC* ^+^ cluster lacking *TRAV*/*TRBV* transcripts differentially expressed *FCER1G*, *KLRC2*, *KIR2DL4*, *KLRD1*, *GNLY* (Granulysin), *PRF1* (Perforin), *XCL1*, *GZMA* and *GZMB* (Supplementary table 2), indicating an NK cell phenotype (Figure 2b, Supplementary table 2). In support of this, a weighted gene co‐expression network analysis (WGCNA) module of 25 transcripts up‐regulated by this cluster was associated with several NK‐related GO terms (GO: 0002717; GO: 0042269; GO: 2000503) (Supplementary table 3). Re‐clustering identified two sub‐populations that could be differentiated by *IFNG* and additional genes reported to delineate human adaptive‐like NK cells^27^ (Supplementary figure 3c, Supplementary table 4). The final sub‐population differentially expressed hallmark T‐cell genes, including *CD247*, *TRAC* and *TIGIT*. As the transcription factor (TF) *ZBTB16* (PLZF^28^) was also up‐regulated (and TCR complex genes were up‐regulated in comparison to NK cells), we concluded these were NKT‐like cells with a tissue‐resident (*ITGAE* ^+^ *ITGA1* ^+^ *ITGB7* ^+^) phenotype; although TCR sequencing was not carried out, which could have addressed this possibility (Supplementary figure 3c).

A second cluster lacking *TRAV*/*TRBV* (*TRDC* ^+^ with reduced TCR complex gene expression) differentially expressed ILC‐associated TFs: *KIT*, *ZBTB16*, *RORC*, *NFIL3*, *AHR*, *RUNX3* as well as *MITF* (bHLHe32) (Supplementary figure 3d, Supplementary table 2). The cluster was mostly closely related to Tc22 cells, and also up‐regulated *IL17F*, *IL17A*, *IL2RA*, *GNG4*, *OLFM4*, *IRF8*, *IL22*, *LTA*, *IL41L*, *FURIN*, *CXCL8* and *CCR6*, indicating a primarily ILC3 phenotype (Figure 2b). We found these cells to up‐regulate genes involved in tyrosine and phenylalanine metabolism, while amino acid availability is known to act as a metabolic rheostat regulating the magnitude of ILC2 responses^29^ (Supplementary figure 3e). Recent studies in paediatric and adult inflammatory bowel disease patients have reported ILC3 frequency to be inversely correlated with endoscopic score, while ILC2s were positively correlated with worsening disease.^30^ We did not detect ILC2‐like cells, supporting a clinically normal state in this animal, although heterogeneous TF usage in less differentiated cells was observed.

### Recent thymic emigrant‐like cells contribute to the caecal T‐cell pool

**Figure 3:**
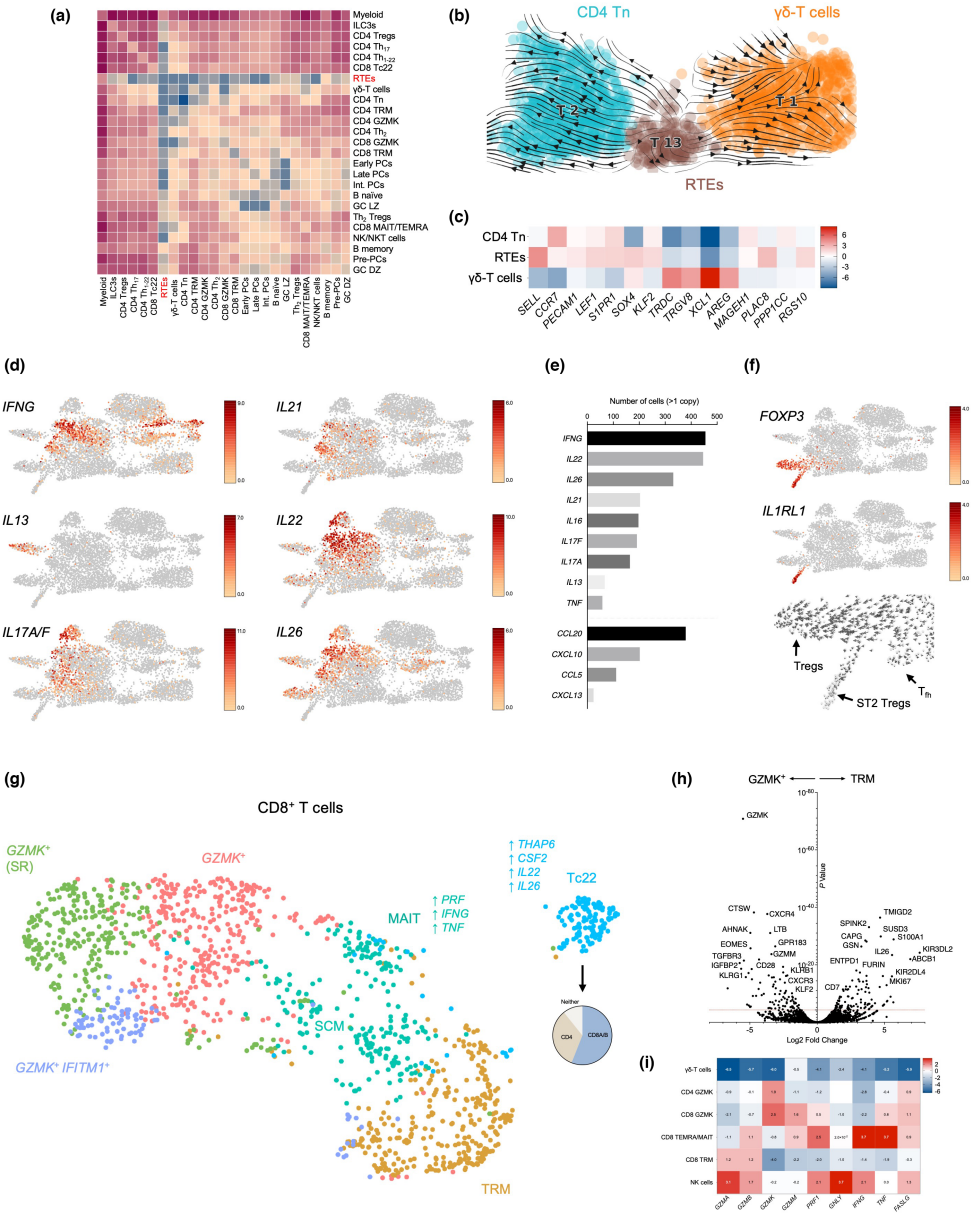
CD4^+^ and CD8^+^ T‐cell effector phenotypes of the rhesus caecum. **(a)** Cell–cell contact (ligand–receptor interaction) analysis performed using CellPhoneDB is shown for all clusters in the dataset. The lower interaction scores for RTEs are highlighted by red text on the axis. **(b)** RNA velocity analysis of RTE, CD4 Tn and γδ‐T‐cell clusters. **(c)** Log_2_ fold‐change of key markers delineating RTEs, CD4 Tn and γδ‐T cells. **(d)** Expression of CD4 T_h_ cytokines amongst T cells and ILCs. Log_2_ expression for each marker is indicated by the scale bar. **(e)** The number of cells expressing (> 1 copy) of T_h_ cytokines and chemokines among CD4 T_h_ cells. **(f)** Expression of FOXP3 and IL1RL1 is shown in the T‐cell and ILC UMAP. The bottom panel illustrates RNA velocities in the Treg region. Log_2_ expression for each marker is indicated by the scale bar. **(g)** Re‐clustering of all CD8 T‐cell populations, illustrating two major subsets – GZMK+ and TRM. Key genes differentially expressed by Tc22 and MAIT cells are shown, as is the proportion of the TC22 cluster expressing either CD4 or CD8A/B. The relative proportions of all CD8 subsets are shown in the bottom panel. SR, stress response. **(h)** Genes differentially expressed by CD8 TRM versus CD8 GZMK+ subsets. **(i)** Log_2_ fold‐change in expression of cytotoxicity molecules among selected T‐cell subsets, highlighting the lower cytotoxic potential of γδ‐T cells and GZMK^+^ subsets.

A small cluster of T cells (3%) differentially expressed *SELL* (CD62L), *SOX4*, *S1PR1* and *LEF1* and occupied a lone branch of transcriptional and metabolic module clustering (Figure 2b, Supplementary figure 3e, Supplementary table 2). These cells had uniquely low cell–cell contact scores based on ligand–receptor interactions analysed using CellPhoneDB,^31^ indicating an un‐differentiated state (Figure 3a). The cluster was metabolically quiescent and up‐regulated nitrogen metabolism genes (Supplementary figure 3e). CD4^+^, *CD8A/B* ^+^, *CD4* ^−^ *CD8A/B* ^−^, *TRAV* ^+^, *TRBV* ^+^, *TRDC* ^+^ and T‐cell receptor gamma (*TRGV*)^+^ cells were observed, suggesting a mixed T‐cell ontogeny. RNA velocity analysis illustrated these cells were on trajectories towards adjacent clusters of differentiating CD4^+^ T cells and γδ‐T cells (Figure 3b and c, Supplementary table 2). Based on these observations and the known roles for *SOX4*,^32^ *LEF1* ^33^ and *S1PR1* ^34^ in thymocytes and recently egressed T cells,^35^ we concluded these were RTE‐like feeding the caecal T‐cell pools. This is consistent with thymic output in aged rhesus macaques, and human and murine studies showing re‐population of the intestinal barrier by RTEs.^36^, ^37^, ^38^, ^39^ Alternatively, the murine thymus has been found to export a population of long‐lived T‐cell precursors that colonise lymphoid follicles, including those in the gut.^40^ It was not possible to address the ontogeny of these cells in this study.

### SOX4^+^ Vγ8Vδ1‐T cells display a tolerogenic phenotype

A cluster comprising ~11% of T cells and ILCs differentially expressed *SOX4*, *ENSMMUG00000057791* (*TRDC*), *ENSMMUG00000054501* (a human *TRGV* orthologue), *ENSMMUG00000060606* (a human *TRGV* orthologue), *XCL1*, *KIR2DL4*, *ID3*, *CRTAM*, *CD7*, *AREG* (Amphiregulin) and *GZMM* (Figure 2a, Supplementary table 2). The cluster showed the highest relative expression of galactose metabolism genes of all T cells and ILCs, as well as elevated OXPHOS gene expression (Supplementary figure 3e). Based on differential expression of *TRDC*, *TRGV* orthologues and lineage TFs (*SOX4*,^41^ *ID3* ^42^ and *TCF7* ^43^), we hypothesised these were γδ‐T cells. To validate this, we explored *TRD* and *TRG* transcripts in more detail. By aligning short reads from 10X against rhesus macaque *TRDV* and *TRGV* databases using kallisto,^44^ we found TRDV1*01 transcripts in the γδ‐T‐cell cluster that were absent from ILC3s and NK cells (Supplementary figure 4a, b). Using the same approach, TRGV8*01 was determined to be the dominant Vγ used (Supplementary figure 4c).

*CCR7* was differentially down‐regulated by γδ‐T cells (Supplementary table 2). In‐keeping with a role related to the epithelial barrier (i.e. *AREG*, *XCL1*, *GZMM*), cell–cell contact analysis showed lower interaction scores for γδ‐T cells compared to most other non‐PCs (Figure 3a). Given the relatively low levels of *IL17A/F* and *IFNG* expression, the data implicate *SOX4* in the development of thymic‐derived Vγ8δ1‐T cells with a tolerogenic phenotype.^45^

### Adult caecal tissue hosts heterogeneous CD4^+^ T helper cells

Another cluster containing RTE‐derived trajectories consisted of predominantly *CD4* ^+^ T cells with similar low expression of OXPHOS genes, and differentially expressing *MAGEH1*, *S1PR1*, *CCR7*, *CR1* and *CR2*, which we termed CD4^+^ T naïve (Supplementary table 2). The *KLF2* target gene, *S1PR1*, also differentially expressed by RTEs, maintains the mitochondrial content and survival of naïve T cells,^46^ while *KLF2* was also expressed by cells in this cluster (Figure 3c). Furthermore, we and others have shown that human CD4^+^ RTEs in human peripheral blood express *CR1* and *CR2*,^47^ which aid naïve T‐cell activation, although these transcripts were here lacking from the less differentiated RTEs, supporting that some CD4^+^ T cells become increasingly responsive to antigen after arriving to the tissue (Supplementary table 2). Re‐clustering identified a sub‐population differentially expressing *KLRB1* and *CXCR3*, indicative of activation (Supplementary table 5).

The majority of remaining CD4^+^ T cells displayed effector‐memory phenotypes, and apart from Tregs, mostly down‐regulated *CCR7* (Supplementary table 2). A prominent cluster of *CCR6*‐expressing *CD4* ^+^ cells was marked by up‐regulation of *IL22*, *CXCL10*, *IFNG*, *IL26*, *CD40LG*, *SOD2*, *CSF2*, *NFKBIA*, *ICOS*, *CD69*, *CTLA4* and *TRAF1*, suggesting a mixed T helper (Th)_1/22_ phenotype (Figure 3d, Supplementary table 2). Re‐clustering revealed two major sub‐populations that expressed similar levels of *IFNG* but could be differentiated by *IL22*, indicating relatedness between Th_1_ and Th_22_ phenotypes^48^ (Supplementary table 6). *IL26* was also strongly up‐regulated by cells in this cluster (Figure 3d, Supplementary table 2).

Th_17_ cells were primarily located in a cluster with robust *IL21* expression that also up‐regulated *CCL20*, *CTLA4*, *CCR6*, *CD28*, *IL17A* and *IL17F* (Figure 3d, Supplementary table 2). Indicative of T_h_ plasticity,^49^ we observed Th_17_ phenotypes in the Th_1‐22_ cluster and *vice versa*, as well as overlapping phenotypes. We concluded that CD4^+^ T‐cell‐derived IL‐21 balances T_h_ and Treg differentiation^50^ and note expression of *CCL20* by *IL21* ^+^ cells, suggesting cells in this cluster may have played a role in the recruitment of *CCR6* ^+^ cells (Supplementary table 2).

With a similar metabolic profile to T_h_ cells, a cluster differentially expressed *CXCR3*, *GZMA*, *ITGA1*, *CD96* (Tactile) and *KLRB1*, compared to other CD4^+^ effectors (Supplementary table 7). These cells transcribed relatively lower levels of cytokines than the activated T_h_ subsets and we concluded these were CD4^+^ Th‐like cells with a tissue‐resident memory (*ITGA1* ^+^ *CXCR3* ^+^ *KLRB1* ^+^) phenotype (Supplementary table 7).

A distinct cluster of CD4^+^ T cells (comparable in size to ILC3s) differentially expressed *IL13*, *GATA3*, *IL17RB*, *PPARG*, *TNFSF11* (RANKL), *TNFRSF11A* (RANK), *IL9R*, *IL17RB* and *PER3*, supporting a Th_2_ phenotype^51^, ^52^ (Figure 3d, Supplementary table 2). Based on these results, it is tempting to speculate that RANKL–RANK signalling tunes caecal IL‐13 responses, as occurs in osteoclasts.^53^ In support of this, *IL13*, *PPARG*, *TNFSF11* and *TNFRSFA11* were in the same WGCNA module up‐regulated by this cluster that was associated with ‘positive regulation of prostaglandin secretion’ (GO:0032308) and ‘positive regulation of fever generation’ (GO:0031622) (Supplementary table 3). Illustrative of their effector functions, this cluster differentially expressed *CYSLTR1*, *ALOX5AP* and *HPGDS* (Supplementary table 2). Th_2_ cells showed the highest expression of the glucocorticoid receptor, *NR3C1*, and also up‐regulated *EPAS1* (HIF‐2α; Supplementary table 2). Notably, a loss of IL‐13 expression by colonic T cells has been associated with chronic diarrhoea in rhesus macaques with idiopathic colitis,^54^ supporting a homeostatic role for these cells, although it is not possible to rule out that the animal had an ongoing Th_2_‐driven response. The abundance of T_h_ cytokines and chemokines is shown in Figure 3e.

### Two distinct FOXP3^+^ Treg populations maintain tolerance

Given the Th_2_ cells, it was interesting to observe two *CD4* ^+^ clusters differentially expressing *FOXP3*: Tregs, and a minor population of ST2^+^ (*IL1RL1* ^+^) Tregs, the latter responsive to type 2 cytokines (Figure 3f, Supplementary table 2). Expression of *IKZF2* (HELIOS), a putative marker of thymic‐derived Tregs, was not significantly different between the two populations, while RNA velocity supported that some Tregs were derived from CD4^+^ T cells, with *FOXP3* expressed by < 1% of RTEs (Supplementary table 2). On the other hand, RNA velocities in ST2^+^ Tregs suggested a more terminally differentiated state (Figure 3f).

The larger of the two Treg clusters (Tregs) differentially expressed *FOXP1*, *FOXP3*, *LAG3*, *IL2RA*, *CCR7*, *CTLA4* and *TIGIT*. Re‐clustering revealed naïve and activated states, the latter resembling caecal T_h_ phenotypes, as well as a small population that differentially down‐regulated *FOXP3* and had features of follicular helper T cells (T_fh_): differential expression of *CD200*, *CXCL13*, *CD40LG*, *BCL2A1*, *BCL6*, *PDCD1*, *FOS*, *ID3*, *CXCR4* and *TOX2*,^55^ indicating overlap between Treg and T_fh_ phenotypes^56^ (Supplementary figure 5a, Supplementary table 8). *CXCR5* was not widely expressed in T cells, being enriched in CD4 Tn and Treg clusters (Supplementary figure 5a, b, Supplementary table 2).

Notably, Treg *CCR7* up‐regulation suggests lymphoid homing capacity, while ST2^+^ Tregs down‐regulated this gene (Supplementary figure 5c). ST2^+^ Tregs differentially expressed *IL1RL1* (ST2; IL‐33R), *CGA*, *GITR*, *OX40* and *GATA3* (Supplementary figure 5d, Supplementary table 2). Like Th_2_ cells, ST2^+^ Tregs up‐regulated the circadian clock gene *PER3*, supporting a periodic element to CD4^+^ type‐2 responses, in agreement with research in humans and mice^57^ (Supplementary tables 2 and 9).

### EOMES^+^GZMK^+^ CD4^+^ T cells are analogous to CD8^+^ counterparts

A population of *CD4* ^+^ T cells clustered alongside *GZMK* ^+^ CD8^+^ T cells and differentially expressed *GZMK*, *IL10*, *MAMU‐DRB1*, *MAMU‐DRA*, *CCR2*, *CCR3*, *CCR5*, *CXCR4*, *CXCR3* and *EOMES* ^58^ (Figure 2a, Supplementary figure 5e, Supplementary table 2). Unlike other activated *CD4* ^+^ cells, these down‐regulated *CCR6* and expressed relatively higher levels of MHC class II genes (Supplementary figure 3d, Supplementary table 2). The cluster had a non‐cytotoxic profile and Granzyme K has been reported to induce pro‐inflammatory and wound healing responses in fibroblasts and epithelial cells without activating apoptotic caspases,^59^ supporting these cells had a role in tissue remodelling, as reported for human GZMK^+^ CD8^+^ T cells in colorectal tumours.^60^ IL‐10^+^ GZMK^+^ CD4^+^ T cells have primarily been described in tumours, as well as nasal polyps from chronic rhinosinusitis patients.^61^, ^62^ Our data indicate a role for this cell type in the caecum.

### GZMK^+^ and TRM CD8^+^ lineages use distinct co‐stimulatory pathways

In total, there were four clusters of CD8^+^ T cells, most of which contributed to *GZMK* ^+^ and TRM populations (Figure 3g). However, a smaller cluster differentially expressed *PRF1*, *CCL4*, *CCL3*, *TNF*, *IFNG* and *BHLHE40* (Figure 3g, Supplementary table 2). A sub‐population of these up‐regulated *TRDC* and several pro‐inflammatory and cytotoxic molecules, as well as *IL2RB*, *IL18RAP* and *IL23R*, supporting that these were innate‐like T cells (Supplementary table 10). In agreement, projection of our dataset onto a human PBMC reference using Azimuth^63^ identified MAIT cells in this region, although TCR sequencing is required to address this possibility (Supplementary figure 5f). The remaining cells showed a resemblance to CD8^+^ TEMRA cells defined in the human lung, and we named the cluster MAIT‐TEMRA.

A second small, predominantly *CD8A*/*B* ^+^ cluster corresponded to Tc22 cells (Figures 2a and 3g). Compared to other CD8^+^ T cells, these differentially expressed *THAP6*, *CSF2*, *IL22*, *IL26* and *CCR6*, with cytokines and *CCR6* transcribed by *CD8B* ^+^ cells (Supplementary table 2). Because of the size of the cluster this was not resolved in further, although previous studies have identified a similar population in atopic dermatitis, cancer and HIV infection.^64^, ^65^

The largest cluster of CD8^+^ T cells was marked by differential expression of *GZMK*, *GZMM*, *GZMB*, *EOMES*, *PRF1*, *KLRG1*, *KLRD1*, *NKG7* and *CRTAM* (Figure 3g, Supplementary table 2). *GZMK* ^+^ CD8 T cells have been reported to form a core population in different human tissues and have low cytotoxic potential.^58^, ^66^ In agreement, we found these cells to differentially express *GZMB* and *PRF1* at the global level, although the expression was decreased compared to NK cells, NKT cells, CD8 TRM and MAIT/TEMRA cells, while *GZMM* was up‐regulated (Figure 3h and i). As observed in their *CD4* ^+^ counterparts, *CXCR4* was up‐regulated and *CCR6* was down‐regulated, although unlike the *CD4* ^+^ population, RNA velocities of *GZMK* ^+^ *CD8* ^+^ T cells supporting these cells could have been in a more terminally differentiated state (Supplementary figure 5g, h).

Alongside up‐regulation of the T‐cell senescence marker, *KLRG1*, the largest sub‐population of *GZMK* ^+^ CD8^+^ T cells showed increased expression of DNA damage and heat shock response/regulation genes, in keeping with a metabolically stressed/exhausted phenotype previously associated with these cells^66^ (Supplementary table 11). However, T‐cell exhaustion markers – *PDCD1* (PD‐1), *TIGIT*, *HAVCR2* (TIM‐3) – were not significantly up‐regulated (Supplementary table 2). The second sub‐population differentially expressed *IL7R*, *SELL*, *LEF1* and *CCR7* (and down‐regulated *GZMB*), resembling a naïve/stem cell memory (SCM) phenotype, while a minor population differentially expressed the interferon response gene *IFITM1* (CD225; Supplementary table 11).

On the other hand, *CD69* ^+^ *ITGA1* ^+^ *ITGAE* ^+^ CD8^+^ TRM cells resembling the human phenotype,^67^ differentially expressed *TMIGD2* (CD28H), *TESC*, *CCL5*, *GZMB*, *GZMA*, *ENTPD1* (CD39), *MAMU‐DRB1*, *KLRD1*, *IL10*, *PTPN22*, *ETS2* and *MKI67* (Figure 3h and i, Supplementary table 2). CD8^+^ TRM expressed the highest levels of *GZMB*, *ITGAE*, *CD101* and *ITGB7* of all T cells, supporting a frontline cytotoxic role at the epithelial barrier, and had a metabolic profile most similar to γδ‐T cells (Supplementary figures 3e and 5i, Supplementary table 2). Notably, CD8^+^ TRM differentially down‐regulated *LTB* [lymphotoxin‐beta, expressed by 68% (> 1 copy) of T cells and ILCs] (Supplementary table 2).

Almost uniquely among T cells and ILCs, a sub‐population of TRM was enriched for cells expressing *MKI67*, supporting their turnover (Supplementary table 12). Indeed, CD8^+^ TRM cells differentially expressed transcripts corresponding to *TRAV* and *TRBV* genes, while recent studies have reported increased clonality of these cells as they replicate at different tissue sites^68^ (Supplementary table 2). Therefore, *TMIGD2* (CD28H, the ligand of HHLA2) serves as a discriminator of TRM compared to *GZMK* ^+^ CD8^+^ T cells in the caecum, with the latter differentially expressing *CD28*. The data support the use of distinct co‐stimulatory pathways by the two CD8^+^ populations, meriting functional investigation at the protein level and *in vivo* (Figure 3h, Supplementary table 2).

### Memory B cells up‐regulate antigen‐presenting genes in caecal patches

**Figure 4:**
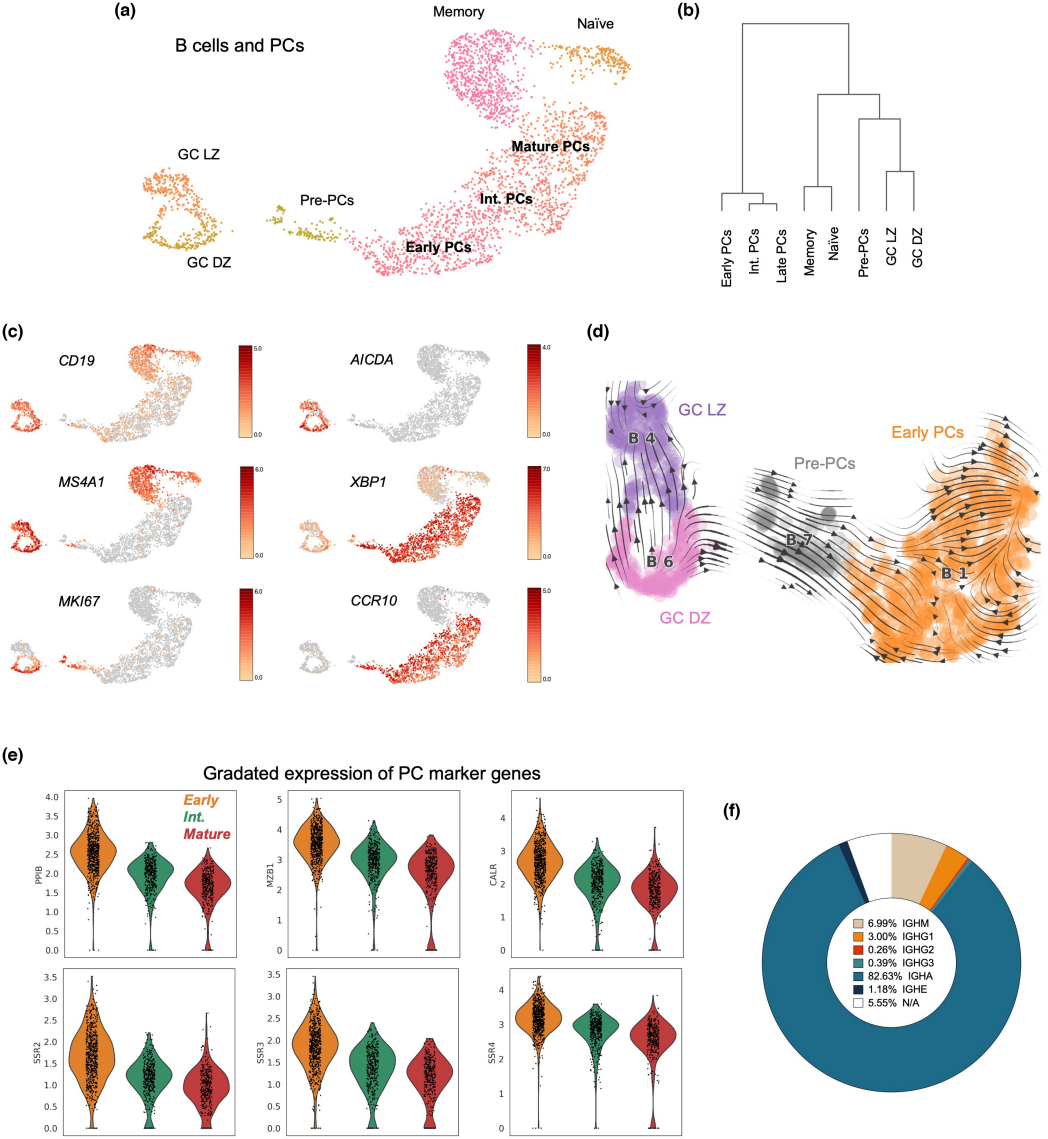
B‐cell phenotypes and PC development in the caecum. **(a)** UMAP of re‐clustered B lineage cells, showing the proportion of follicular and plasma cell populations (inset pie chart). **(b)** Clustering analysis of B lineage cells based on 3000 highly differentially expressed genes. **(c)** Expression of key B lineage markers is shown. Log_2_ expression for each marker is indicated by the scale bar. **(d)** RNA velocity analysis of GC B cells, pre‐PCs and early PCs, showing GC DZ cells on a trajectory towards the PC phenotype. **(e)** Expression gradients of key PC genes are shown for early, intermediate and mature PC subsets. **(f)** Paired, full‐length caecal PC antibodies according to isotype.

In total, eight clusters of B lineage cells were identified (Figure 4a and b). These consisted primarily of follicular (CP) B cells, GC B cells and PCs, which could be distinguished from each other by *CD19*, *MS4A1*, *MKI67*, *AICDA*, *XBP1* and *CCR10* expression (Figure 4c, Supplementary figure 6a).

Naïve B cells differentially expressed *SELL*, *S1PR1*, *KLF2*, *KLF3*, *FCER2* (CD23), *CR1*, *CR2*, *CD9*, *CCR7*, *VPREB3*, *FCMR* and *IL10RA* (Supplementary table 13). Unlike PCs, these cells showed high expression of *CCR7*, *CXCR4* and *CXCR5*, suggesting localisation to caecal patches. The adjacent population (25% of B lineage cells) differentially expressed *SIGLEC6*, *CCR7*, *BCL2A1*, *CD52*, *CD86*, *ZBTB32*, *CXCL10*, *ITGAX*, *MAMU‐DRA*, *MAMU‐DPA1*, *MAMU‐DRB1*, *BHLHE40*, *CXCR5*, *CXCR4* and *LTB* (Supplementary figure 6b, Supplementary table 2). As metabolic genes were also up‐regulated compared to naïve cells, we concluded these were primarily follicular memory B cells (Supplementary figure 6c, d). Apart from myeloid cells, these cells had the highest levels of MHC class II gene expression in the dataset and differentially expressed different WCGNA modules associated with B‐cell activation, lysosome and endosome function, and MHC class II protein complex binding (Supplementary table 3). *CD40* was also strongly differentially expressed, supporting a role in CD4^+^ T‐cell activation. RNA velocity analysis indicated that a proportion of memory B cells were on a trajectory towards a PC phenotype, indicating some may go on to acquire antibody secreting capacity (while the remainder showed lower RNA velocities; Supplementary figure 6e).

### Continual PC generation is associated with gradated lineage signatures

Two distinctive B‐cell populations strongly differentially expressed activation‐induced cytidine deaminase (*AICDA*; AID, *P* ‐ 4.04 × 10^−116^) – typical of GC B cells undergoing somatic hypermutation (SHM) – as well as *RGS13* (*P* – 3.76 × 10^−131^; Figure 4c, Supplementary table 13). We termed these light zone (LZ) and dark zone (DZ) GC B cells based on the differential expression of the proliferation antigen, *MKI67*, by DZ cells (Figure 4c, Supplementary table 13). This was supported by elevated metabolic gene expression in DZ versus LZ B cells, and the expression of WGCNA modules associated with mitosis, cytoskeletal re‐organisation and V(D)J recombination by DZ cells (Supplementary table 3).

RNA velocity analysis showed DZ GC cells were on a trajectory towards pre‐PCs and early PCs (Figure 4d). DZ trajectories also illustrated some cells were on a path towards the LZ, potentially for further BCR selection. The small cluster of pre‐PCs differentially expressed *MKI67* and had a similar metabolic profile to GC cells, but lacked *AICDA*, indicating they had finished SHM (Figure 4c, Supplementary table 13).

To obtain information about IG heavy (H) and light (L) chain V(D)J transcripts, we incorporated IG constant region primers into the experiment. PCs expressing paired, full‐length IG transcripts comprised 52% of B lineages and 16% of the total dataset, corresponding to three clusters. Globally distinguishing PC features included *CCR10*, *TNFRSF17* (BCMA), *SDC1* (CD138), *MZB1*, *DERL3*, *PRDX4*, *XBP1*, *CD63*, *CD160*, *CD59*, *ICAM2* and *CALR*, as well as insulin‐like growth factor 1 (*IGF1*; Supplementary table 14). Unlike other B lineage cells, PCs were negative for *CCR7*, *CXCR4* and *CXCR5*, and showed robust expression of *ICAM1*, *ICAM2*, *ICAM3*, *ITGB7*, *ITGAE* and *ITGAL*, suggesting that these molecules modulate their retention (Supplementary table 14). Notably, *CADM1* (a ligand for *CRTAM*) and *CD160* were also differentially expressed and are known to maintain LP and intra‐epithelial T cells.^69^

Across the three clusters of PCs, gradated expression of several lineage and metabolic genes was observed, with the highest levels observed in early PCs (Figure 4e). Based on these expression gradients and the decreasing proportion of each PC subset captured, we named them as early (694 cells), intermediate (436 cells) and mature (369 cells) subsets. In‐keeping with their more robust transcription, early PCs expressed a WCGNA module associated with protein processing and export from the ER that was not observed in intermediate and mature subsets (Supplementary table 3). Together, the data support a model in which continual B‐cell proliferation is required to maintain a PC pool of limited lifespan, in agreement with murine studies showing *Ccr10* ^+^ PCs turn over more quickly than those from other sites.^70^ PC longevity in different tissues and contexts is a topic of great interest and could not be addressed by the methods applied.

### Affinity‐matured caecal PCs lineages can be traced in different tissues

To obtain highly precise gene assignments and clonotype data, we generated individualised IGH, IGK and IGL genotypes for H03 using RepSeq and IgDiscover.^71^ Paired heavy and light chain V(D)J sequences (1531 antibodies) obtained from single cells illustrated that most caecal PCs (83%) expressed IgA molecules. These were followed by IgM (7%), IgG_1_ (3%), IgE (1%), IgG_3_ (0.3%) and IgG_2_ (0.3%), while 6% were of unknown isotype (Figure 4f). Our data indicate an important contribution from unswitched memory at this site, while low‐frequency IgE responses could reflect steady‐state type 2 immunity in the macaque.

**Figure 5:**
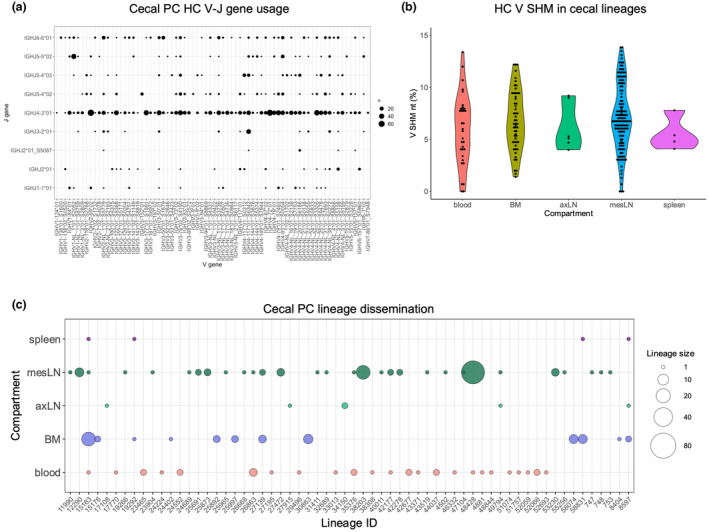
The caecal PC antibody repertoire disseminates throughout the body. **(a)** Number of sequences corresponding to different heavy chain V and J genes is shown, illustrating a dominant role for IGHJ4‐3*01 within the polyclonal repertoire. **(b)** Heavy‐chain V gene SHM at the nucleotide level for caecal clonotypes traced in different compartments. axLN, axillary lymph nodes; mesLN, mesenteric lymph nodes. **(c)** Caecal clonal lineages traced in different compartments according to their relative abundance.

Heavy chain V and J genes usage was comparable to that found in IgG and IgA repertoires deep sequenced from the peripheral blood, spleen, mesenteric lymph node and bone marrow, consistent with previous research^72^ (Figure 5a, Supplementary figure 7a, b). A total of 656 antibody lineages were identified among the 1531 caecal PC antibodies (Figure 5b, Supplementary table 15). To determine whether members of these lineages had disseminated to different tissues, we traced these in IgG/IgA libraries from peripheral blood, spleen, different lymph nodes and bone marrow. We found caecal lineages were most abundant in the mesenteric lymph node and peripheral blood, while they were largely absent from the spleen and axillary lymph nodes (Figure 5c). Tracing in bone marrow samples suggest cells of caecal origin may contribute to the PC pool residing in the femur (Figure 5c). The results support that ongoing PC generation in caecal patches contributes systemic antibody responses.

## Discussion

Molecular characterisation of immune cells from different anatomical compartments furthers understanding of organism and tissue development, remodelling and defence.^73^ Recent studies in humans using single‐cell and spatial transcriptomics have shown similarity between the human large intestine and many of the cell types here described,^3^, ^4^ although the human caecum and appendix remain to be described by similar studies. Human studies indicate that the ascending, transverse and descending colon are broadly similar to each other in terms of adaptive immune cell types and proportions, with prominent roles for CD4^+^ and CD8^+^ T cells, γδ‐T cells and plasma cells in hierarchies similar to this macaque.

As in the human large bowel, our data support ILC3s are an important source of IL‐17 in the healthy caecum and here lacked *IFNG* expression observed during simian immunodeficiency virus (SIV)‐mediated chronic inflammation.^74^ As the Th_1_–Th_17_ balance has been implicated in the progression from SIV to AIDS and bacterial translocation,^75^ ILC3s should be considered in this dynamic. More generally, *TRDC* expression by ILCs merits further exploration, as it makes it tempting to speculate that mechanisms at the *TRD* locus bifurcate invariant T cells and ILCs during development.^76^

In terms of T cells, our results support that RTEs (or a population of T‐cell progenitors) contribute to the intestinal T‐cell pool in macaques, as reported in humans and mice.^38^, ^39^ However, their precise ontogeny, TCR rearrangements, number/frequency according to age, and which cells they go on to generate, will need to be determined in mechanistic studies, as they could be targeted to bolster intestinal immunity. Thymectomy of juvenile macaques did not result in higher viral loads or faster disease progression after SIV infection, suggesting peripheral T‐cell destruction is the major route to pathology in this context.^77^

Alongside the marked anti‐pathogen responses of NK cells, CD8^+^ TRM and T_h_ cells, which may need to be tempered in severe acute or chronic inflammation, the data highlight a prominent role for T cells in epithelial maintenance and remodelling. Vγ8Vδ1‐T cells transcribed *AREG* and *XCL1*, two γδ‐T‐cell‐derived factors found to maintain tolerance at barrier sites,^78^, ^79^, ^80^ while T_h_, Tc22 and ILC3s expressed epithelial/stromal‐trophic *IL22*, currently being modulated in several clinical trials (https://clinicaltrials.gov). In addition, subsets of CD4^+^ and CD8^+^ T cells could be delineated by non‐apoptotic *GZMK*. Such responses likely help balance pro‐inflammatory responses to maintain barrier integrity, and their TCR specificities will be interesting to define. Another example of such a possibility is the presence of ST2^+^ Tregs. IL‐33 and *GATA3* are mediators of type 2 immunity and ST2^+^ Tregs have previously been defined in the large intestinal mucosa, adipose and other tissues of humans and mice, where they sense the epithelial alarmin (IL‐33) to mediate anti‐inflammatory and metabolic effects.^81^, ^82^ Genetic studies in mice illustrated that ST2^+^ Tregs depend on a *Sted2d*‐*Gata3* axis to suppress Th_2_ cells during colitis.^83^

With regard to other CD4^+^ T_h_ cells, and unlike what has been generally described in clean laboratory mice (often 6–12 weeks of age), TF and effector gene usage in the caecum was more heterogeneous. This suggests that T_h_ cells adopt plastic phenotypes, although archetypal T_h_ signatures were visible at the global level. Interestingly, CD4^+^ T cells showed higher RNA velocities than CD8^+^ T cells.

*IFNG* and *IL26* CD4^+^ responses stood out in terms of abundance, while anti‐IL‐26 monoclonal antibodies have shown promise in animal models of chronic inflammation.^84^ IL‐26 was also up‐regulated by CD8^+^ TRM cells.

We found CD8^+^ T cells to primarily bifurcate along two lineages – *GZMK* ^+^ and TRM. The former *EOMES* ^+^ population up‐regulated *KLRG1* and had lower expression of metabolic genes – despite transcribing relatively high levels of specific effector molecules. GZMK^+^ CD8^+^ T cells have been reported to regulate intestinal epithelium cells in response to neutrophil activation in tumours,^60^ and our data are consistent with them adopting a primarily non‐cytotoxic role. TRM on the other hand were primarily cytotoxic effectors differentially expressing *TMIGD2* and lacking *CD28*, illustrating distinct effector and co‐stimulatory requirements that, if validated at the protein level, could be targeted *in vivo*.

Globally, the dataset illustrates a higher rate of B‐cell versus T‐cell proliferation in caecal tissue. A substantial proportion of B cells corresponded to follicular memory B cells, which may have arisen through local GC reactions.^85^ The data support memory B cells could have played a role in maintaining class II‐dependent T‐cell responses in caecal patches, while the continual generation of PCs engenders antibodies the specificities of which will need to be determined to further understand microbiome, pathogen and allergen regulation in different populations. Interestingly, IGF‐1 has previously been found to be a major growth and migration factor for myeloma cells^86^ and our data showed up‐regulation of this gene by mucosal PCs.

Antibody lineage tracing indicated that caecal PC clonotypes distributed systemically, primarily to the mesenteric lymph node, peripheral blood and bone marrow. Our data are consistent with a continual need for caecal PC replenishment, which may accommodate temporal changes in gastrointestinal antigens. In this respect, it is notable that a wide range of foods engender peripheral antibody responses.^87^ Therefore, further analyses of gut Th_2_, ST2^+^ Treg and IgE^+^ PC responses (and cell types and gene networks that could arise because of them, e.g. ILC2s) may lead to insights for managing the growing problem of food allergies.^88^

Our data of a highly active immune barrier encourages the collection of caecal biopsies, blood samples and epidemiological information from individuals with acute and chronic diseases affecting the site. For example, molecular analysis of ‘patch lesions’ and healthy tissue from IBD patients may pin‐point dysregulated, druggable pathways, while cohort studies testing whether caecectomy or ileocecectomy (often carried out to reduce complications from appendicitis or during tumour resection) impact immunophenotypes, could also further describe the role of the tissue. While anatomically very different, studies of mice lacking the tissue have shown long‐term alterations in gut bacterial community structures,^89^ as well as an impaired ability to recover from *Citrobacter rodentium* challenge.^90^ Similar studies of primates have not been described, although appendectomy and colectomy have been associated with systemic differences in immune parameters, as well as increased communicable and non‐communicable disease risk, meriting further investigation.^91^, ^92^

Together, this study provides a useful resource for the consideration of primate caecal immunity.

### Study limitations

This research was carried out in primary cells from a single individual. Further research is required to understand how the phenotypes reported vary between individuals according to biological (e.g. genetic, age, sex) and environmental (e.g. diet and microbiota) variables, as well as how mRNA profiles translate to protein phenotypes and contribute functional and cellular effects. This animal was previously enrolled in candidate vaccine studies. Therefore, it is not possible to exclude that immunisation impacted observations.

## Methods

### Animal studies

The rhesus macaque of Chinese origin (H03, 5‐year‐old female) used in this study was housed at the Astrid Fagraeus Laboratory at Karolinska Institutet. Housing and care procedures complied with the provisions and general guidelines of the Swedish Board of Agriculture. The facility has been assigned an Animal Welfare Assurance number by the Office of Laboratory Animal Welfare (OLAW) at the National Institutes of Health (NIH, Bethesda, USA). Macaques were housed in groups in 14 m^3^ enriched cages; habituated to the housing for more than 6 weeks before any experiment; subjected to positive reinforcement training to reduce the stress associated with experimental procedures. H03 was confirmed negative for simian immunodeficiency virus (SIV), simian T‐cell lymphotropic virus, simian retrovirus type D and simian herpes B virus. All experiments were carried out in accordance with an ethical permit obtained from the regional Ethical Committee on Animal experiments (Stockholms Norra Djurförsöksetiska Nämnd, Sweden) and in strict adherence of best practice and legislation. H03 was previously enrolled in a SARS‐CoV‐2 vaccine study, weighed 6.43 kg at termination, and showed no clinical signs.

### Sample collection and processing

An ~3‐cm‐long cross‐section of cecal tissue ending at apex caeci was dissected post‐mortem and kept in ice‐cold PBS + 2% FBS (both from Thermo Fisher Scientific, Waltham, USA) until processing. The apex was opened and cleaned of luminal contents before cutting into small pieces and washing twice with vortexing in 10 mL ice‐cold PBS + 10 mm HEPES. Epithelial cells and intraepithelial lymphocytes were removed by incubation in stripping buffer (Gibco RPMI‐1640 + 2% FBS + 10 mm HEPES +1 mm DTT + 5 mm EDTA – all from Thermo Fischer Scientific) at 37°C for 1 h. The underlying tissues were then washed and enzymatically digested for 1 h at 37°C in RPMI‐1640 + 0.42 mg mL^−1^ Liberase (Roche, Basel, Switzerland) + 60 μg mL^−1^ DNase I (Roche) before physically dissociating with forceps and vortexing. Dissociated tissue was passed through a 70‐μm filter and the single‐cell suspension harvested at the interface of a 40/80% (v/v) Percoll (GE Healthcare, Chicago, USA) gradient and washed twice in ice‐cold PBS + 2% FBS before proceeding to downstream applications. The protocol was adapted from Castro‐Dopico *et al*.^93^

### Histology

A portion of the caecal apex was immersed in 4% PFA for 24 h before transferring to PBS + 30% sucrose (w/v) for a further 24 h. Tissue blocks were embedded in OCT medium (Sakura Finetek, Nagano, Japan), frozen and cut into 10‐μm‐thick slices. H&E staining was carried out using standard protocols at the Biomedicum Histology Core Facility (Karolinska Institutet).

### Fluorescence‐activated cell sorting

The single‐cell suspension of lamina propria and caecal patch cells was re‐suspended at 1 million cells mL^−1^ and stained with Invitrogen LIVE/DEAD Fixable Aqua Dead Cell Stain Kit (Thermo Fisher Scientific) for 15 min at 37°C in PBS. After washing with PBS + 2% FBS, labelled cells were stained with anti‐rhesus macaque CD45‐APC (clone D058‐1283; BD, New Jersey, USA) for 30 min at 4°C. Live CD45^+^ cells (200 000) were acquired into a 4‐mL tube using a FACS Aria Fusion (BD) (Supplementary figure 1b). Data were analysed in FlowJo v.10 (BD).

### Single‐cell transcriptomic‐V(D)J sequencing

A total of 200 000 *ex vivo* CD45^+^ leucocytes isolated from the CP‐LP prep by FACS were diluted and used to load one channel of a 10× chip (10X Genomics, Pleasanton, USA). The primers used to amplify macaque IG transcripts are shown in Supplementary table 16. The experiment was carried out at the Eukaryotic Single Cell Genomics facility at SciLifeLab (Stockholm, Sweden) following validated protocols.

### Single‐cell data analysis

Single‐cell data analysis was carried out in Scanpy (v1.9.1) under Python 3.8. In total, 10 487 cell events were collected by the experiment. Through quality control (QC), cells with more than 10% of reads mapping to mitochondrial genes were excluded. Cells with fewer than 1200 UMIs or 500 expressed genes were also removed. We also excluded genes detected in fewer than 10 cells from subsequent analysis. Doublet detection was carried out using Scrublet (v0.2.3), which identified 614 doublet events for exclusion, and additionally, cells expressing more than one IG heavy or light chain were filtered out. After QC, 9241 cell events remained for further investigation. Highly variable genes (HVGs, *n* = 3000) were selected with Seurat v3 method implemented in Scanpy. Expression values were normalised and log transformed before regression analysis and scaling for dimensionality reduction. Clustering analysis was performed using the Leiden algorithm. UMAP projections were then imported to Loupe Browser (10X Genomics) for analysis of differential gene expression and population re‐clustering. Differential expression *P‐*values were adjusted using the Benjamini–Hochberg method and expression data are presented in the Supplementary tables (*P* < 0.05). For annotation purposes, human orthologues of rhesus macaque genes (e.g. ENSMMUG00000057791 – *TRDC*) were downloaded from www.genenames.org/tools/hcop; restricting to features present both in Ensemble and NCBI databases.

#### WGCNA

Highly variable genes were selected for weighted gene correlation network analysis (WGCNA). An adjacency matrix for selected genes was built based on normalised counts and was further used for topological overlap matrix (TOM). The resulting TOM matrix was hierarchically clustered with Ward's linkage to determine 100 gene modules. HVGs, normalised between [0,1] with l2‐normalisation, were grouped by the corresponding module and their expression was averaged within each cell. Each gene module was tested by fitting generalised linear models (GLM) with each identified cell cluster as the explaining variable. The models for each hypothesis evaluated the residual deviances of the fitted model and null hypothesis model. The percent difference between these metrics was utilised to see whether a particular cell cluster affects the expression level of correlated gene expression modules.^26^

#### Cell–cell contact analysis

To identify cell–cell communications mediated through ligand–receptor interactions within disclosed cell clusters we used CellPhoneDB, a publicly available repository of curated receptors, ligands and their interactions in the human system. As the application was initially built for human, we replaced gene IDs with human orthologues for other species and converted the rhesus macaque genes to their corresponding human orthologues, as recommended in the documentation (https://github.com/ventolab/CellphoneDB). To evaluate cell–cell communications, we extracted the normalised count matrix.

#### RNA velocity analysis

RNA velocity analysis was performed with velocyto (v0.17) and scVelo (v0.2.5) Python packages. A loom file with spliced/un‐spliced count matrices was quantified on the CellRanger output folder with velocyto run10x command as the initial step. The resulting loom file was merged into scanpy AnnData objects of T cells and ILCs, as well as B lineage cells, which were analysed separately. Using the scVelo package the spliced and un‐spliced counts were then normalised. The moments were calculated for each cell across its 30 nearest neighbours in the PCA space. The velocities and corresponding velocity graphs were estimated by stochastic modelling of transcriptional dynamics. The velocity vectors were mapped to the UMAP embeddings of B and T cells.

#### Evaluation of TRDV and TRGV abundances

To determine the phenotype of γδ‐T cells we used the kallisto quant algorithm. Following the kallisto index manual, we used the transcriptome file for *Macaca mulatta* from Ensembl and additionally enriched it with V fasta files for TR genes from www.IMGT.org. The unmapped reads were filtered out from pseudoalignments and only reads with posterior probability of alignment higher than 0.8 were further analysed. The accordance of Kallisto output with CellRanger output was checked through by correlating UMI counts for *GZMK* (Supplementary figure 4a). The number of unique UMI for corresponding reads was plotted on UMAP projection for selected TR genes identified in differential expression data. *ENSMMUG00000057791*, *ENSMMUG00000054501* and *ENSMMUG00000060606* were annotated as orthologues of human *TRD* and *TRGV* genes. We aligned them against TR databases and defined that they correspond to macaque *TRDC*, TRGV8*01 and TRGV3*01 respectively.

#### Expression of metabolic and phenotypic genes

Analysis of metabolic gene profiles was done in two complementary ways. Firstly, mean expression of metabolic pathway genes described by NanoString Technologies (Seattle, USA) were plotted for each cluster. Secondly, mean expression of metabolic pathway genes described in the KEGG database were plotted for each cluster. Average expression and Log_2_ fold‐change in expression of mitochondrially encoded OXPHOS genes (*COX1*, *COX2*, *COX3*, *COX4I1*, *COX5A*, *COX5B*, *ND1*, *ND2*, *ND3*, *ND4*, *ND5*, *ATP6*) was used to compare ETC transcript abundance between clusters. The same approach was taken for MHC class I (*MAMU‐A*, *MAMU‐F*) and II (*MAMU‐DOA*, *MAMU‐DOB*, *MAMU‐DMB*, *MAMU‐DRA*, *MAMU‐DRB1*) genes and TCR complex transcripts (*CD3D*, *CD3E*, *CD3G*, *CD247*).

### V(D)J sequence analysis

Bulk IgG/IgA libraries were initially processed with IgDiscover for assignment of the individual V, D and J germline repertoires.^94^ Paired V(D)J sequences obtained from single‐cell analysis were processed using the IgBlast module from IgDiscover for assignment of individual VDJ germline repertoire for heavy chain and rhesus V and J sequences for light chain obtained from IMGT. We then performed denoising processing using the Fast Amplicon Denoising (FAD) tool^95^ and removed chimeric sequences using a hidden Markov model designed for this purpose.^94^ Lineages were identified using the IgDiscover clonotype module by combining heavy chain sequences from single‐cell and bulk sequencing. Lineages were defined by identical V and J allele assignments, identical CDR3 lengths and permitting a maximum of 20% mismatches to nucleotide CDR3 sequences. If single‐cell sequences with different light chain assignments were assigned to the same lineage, the lineage was redefined by also considering same V and J alleles assignment of the light chain and same CDR3 length. In case of divergent light chains, lineage assignment was determined by minimum Levenshtein distance using each single‐cell heavy chain sequence as reference.

## Conflict of interest

The authors declare no competing interests that could have compromised the integrity of this study.

## Author contributions

**Xaquin Castro Dopico:** Conceptualization; formal analysis; investigation; writing – original draft; writing – review and editing. **Mariia Guryleva:** Formal analysis; investigation; methodology; writing – review and editing. **Marco Mandolesi:** Formal analysis; investigation; writing – review and editing. **Martin Corcoran:** Formal analysis; methodology. **Jonathan M Coquet:** Investigation; methodology. **Ben Murrell:** Funding acquisition; investigation; methodology; supervision; writing – review and editing. **Gunilla B Karlsson Hedestam:** Funding acquisition; investigation; resources; supervision; writing – review and editing.

## Data and code availability

All data and code arising from this project are available at https://github.com/MurrellGroup and EMBL‐EBI ArrayExpress (https://www.ebi.ac.uk/biostudies/arrayexpress, accession number: E‐MTAB‐13902).

## Supporting information


Supplementary figures 1‐7

Supplementary tables 1‐16
