# Supplementary material for: Maintenance of caecal homeostasis by diverse adaptive immune cells in the rhesus macaque
Source: Clin Transl Immunology. 2024 May 2;13(5):e1508. doi: 10.1002/cti2.1508 (PMC11063928; doi:10.1002/cti2.1508)
Supplement: Supplementary file 1 — Supplementary figures 1‐7 [file CTI2-13-e1508-s001.pdf]

Supplementary Figure File

Maintenance of cecal homeostasis by diverse adaptive immune cells in the rhesus macaaue

Castro Dopico *et al* 2024

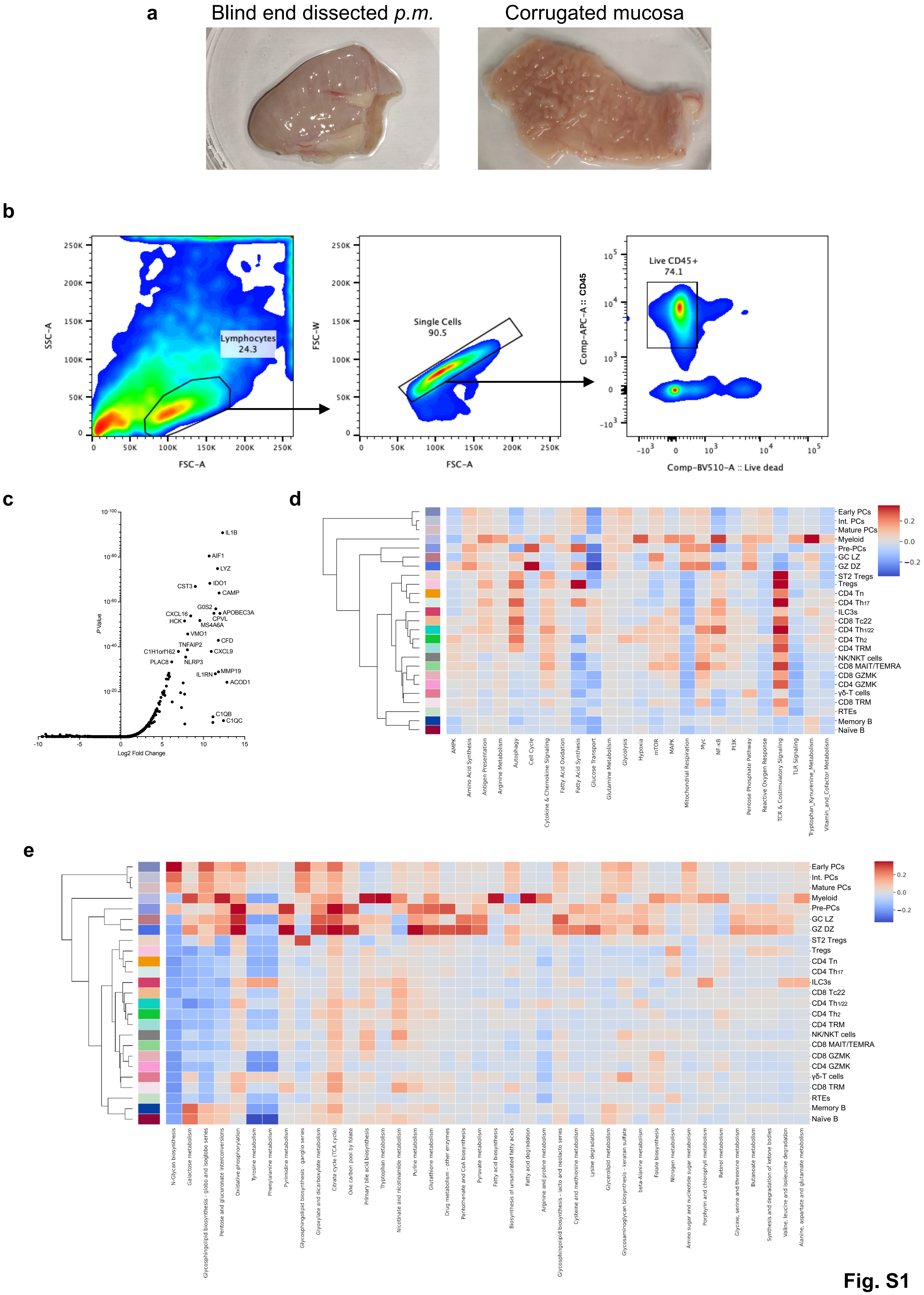

a
Blind end dissected p.m.
Corrugated mucosa
b
Lymphocytes
24.3
SSC-A
FSC-A
Single Cells
90.5
FSC-W
FSC-A
Live CD45+
74.1
Comp-APC-A :: CD45
Comp-BV510-A :: Live dead
c
P Value
Log2 Fold Change
IL1B
AIF1
LYZ
CST3
IDO1
CAMP
G0S2
CXCL16
APOBEC3A
HCK
CPVL
MS4A6A
VMO1
CFD
TNFAIP2
C1H1orf162
CXCL9
PLAC8
NLRP3
MMP19
IL1RN
ACOD1
C1QB
C1QC
d
Early PCs
Int. PCs
Mature PCs
Myeloid
Pre-PCs
GC LZ
GZ DZ
ST2 Tregs
Tregs
CD4 Tn
CD4 Th17
ILC3s
CD8 Tc22
CD4 Th1/22
CD4 Th2
CD4 TRM
NK/NKT cells
CD8 MAIT/TEMRA
CD8 GZMK
CD4 GZMK
γδ-T cells
CD8 TRM
RTEs
Memory B
Naïve B
AMPK
Amino Acid Synthesis
Antigen Presentation
Arginine Metabolism
Autophagy
Cell Cycle
Cytokine & Chemokine Signaling
Fatty Acid Oxidation
Fatty Acid Synthesis
Glucose Transport
Glutamine Metabolism
Glycolysis
Hypoxia
mTOR
MAPK
Mitochondrial Respiration
Myc
NF-κB
PI3K
Pentose Phosphate Pathway
Reactive Oxygen Response
TCR & Costimulatory Signaling
TLR Signaling
Tryptophan_Kynurenine_Metabolism
Vitamin_and_Cofactor_Metabolism
e
N-Glycan biosynthesis
Galactose metabolism
Glycosphingolipid biosynthesis - globo and isoglobo series
Pentose and glucuronate interconversions
Oxidative phosphorylation
Tyrosine metabolism
Phenylalanine metabolism
Pyrimidine metabolism
Glycosphingolipid biosynthesis - ganglio series
Glyoxylate and dicarboxylate metabolism
Citrate cycle (TCA cycle)
One carbon pool by folate
Primary bile acid biosynthesis
Tryptophan metabolism
Nicotinate and nicotinamide metabolism
Purine metabolism
Glutathione metabolism
Drug metabolism - other enzymes
Pantothenate and CoA biosynthesis
Pyruvate metabolism
Fatty acid biosynthesis
Biosynthesis of unsaturated fatty acids
Fatty acid degradation
Arginine and proline metabolism
Glycosphingolipid biosynthesis - lacto and neolacto series
Cysteine and methionine metabolism
Lysine degradation
Glycerolipid metabolism
Glycosaminoglycan biosynthesis - keratan sulfate
beta-Alanine metabolism
Folate biosynthesis
Nitrogen metabolism
Amino sugar and nucleotide sugar metabolism
Porphyrin and chlorophyll metabolism
Retinol metabolism
Glycine, serine and threonine metabolism
Butanoate metabolism
Synthesis and degradation of ketone bodies
Valine, leucine and isoleucine degradation
Alanine, aspartate and glutamate metabolism

Fig. S1

Supplementary Figure 1: Sample processing and metabolic phenotypes

(a) The blind end of the cecum from H03 was photographed before processing. The corrugated mucosa of the cecal blind end is shown in the right-hand panel. **(b)** FACS strategy to isolated $CD45^{+}$ leukocytes. 200,000 live cells were sorted and diluted for genomics. Down-sampled events shown. **(c)** Genes DE by myeloid cells, compared to remaining clusters. **(d)** Mean expression of NanoString metabolic pathway genes in all clusters in the dataset. **(e)** Mean expression of KEGG metabolic pathway genes in all clusters in the dataset.

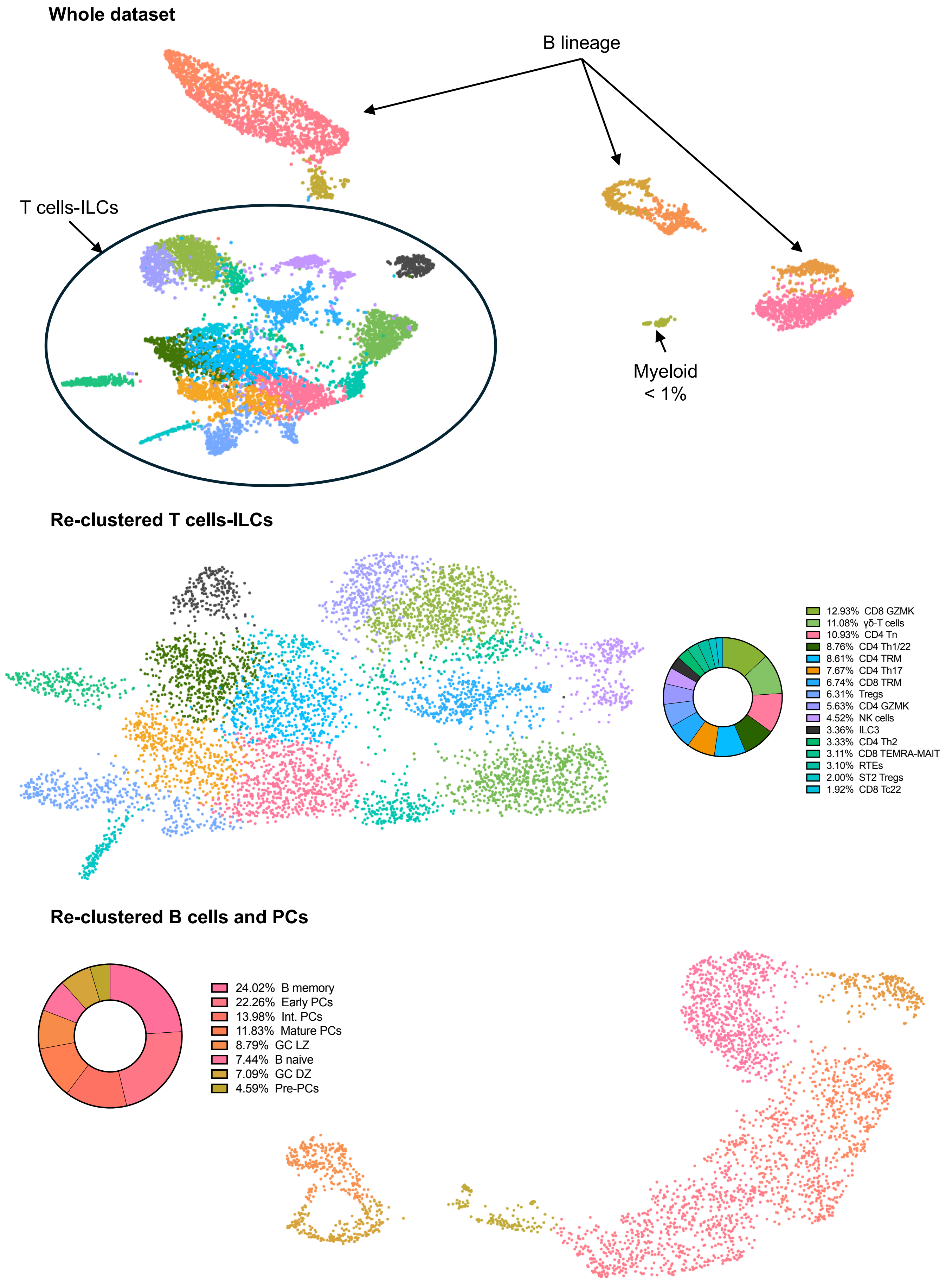
Whole dataset
B lineage
T cells-ILCs
Myeloid
< 1%
Re-clustered T cells-ILCs
12.93% CD8 GZMK
11.08% γδ-T cells
10.93% CD4 Tn
8.76% CD4 Th1/22
8.61% CD4 TRM
7.67% CD4 Th17
6.74% CD8 TRM
6.31% Tregs
5.63% CD4 GZMK
4.52% NK cells
3.36% ILC3
3.33% CD4 Th2
3.11% CD8 TEMRA-MAIT
3.10% RTEs
2.00% ST2 Tregs
1.92% CD8 Tc22
Re-clustered B cells and PCs
24.02% B memory
22.26% Early PCs
13.98% Int. PCs
11.83% Mature PCs
8.79% GC LZ
7.44% B naive
7.09% GC DZ
4.59% Pre-PCs

Fig. S2

Supplementary Figure 2: Dataset re-clustering into T cells and ILCs, and B lineage cells

The whole dataset (top panel) is divided into T cells and ILCs (middle panel) and B lineage cells (bottom panel). Cluster identifies corresponding to colour in the UMAP is inset.

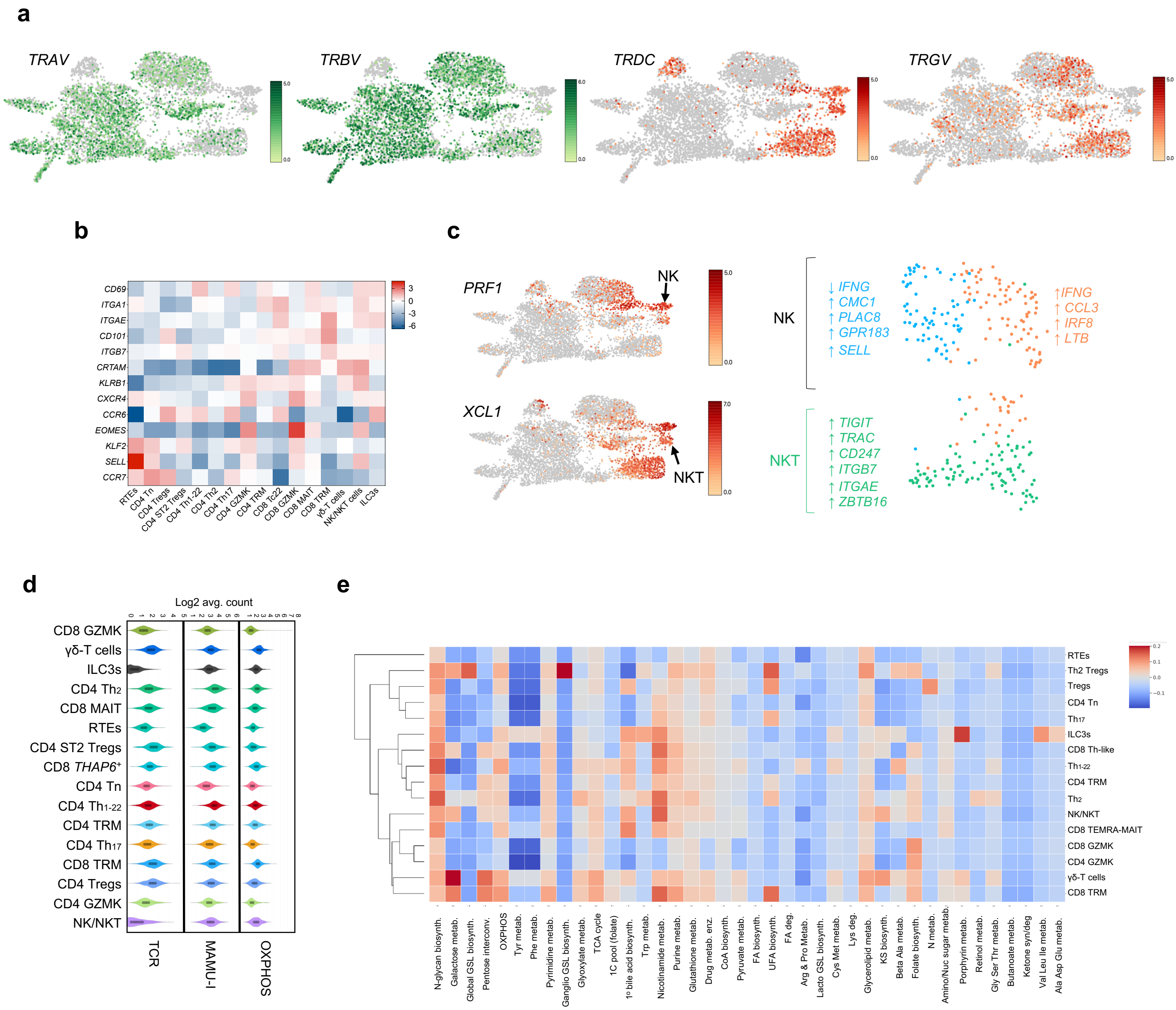
a
TRAV
TRBV
TRDC
TRGV
b
CD69
ITGA1
ITGAE
CD101
ITGB7
CRTAM
KLRB1
CXCR4
CCR6
EOMES
KLF2
SELL
CCR7
RTEs
CD4 Tn
CD4 Tregs
CD4 ST2 Tregs
CD4 Th1-22
CD4 Th2
CD4 Th17
CD4 GZMK
CD4 TRM
CD8 Tc22
CD8 GZMK
CD8 MAIT
CD8 TRM
γδ-T cells
NK/NKT cells
ILC3s
c
PRF1
NK
XCL1
NKT
↓ IFNG
↑ CMC1
↑ PLAC8
↑ GPR183
↑ SELL
↑IFNG
↑ CCL3
↑ IRF8
↑ LTB
↑ TIGIT
↑ TRAC
↑ CD247
↑ ITGB7
↑ ITGAE
↑ ZBTB16
d
Log2 avg. count
CD8 GZMK
γδ-T cells
ILC3s
CD4 Th2
CD8 MAIT
RTEs
CD4 ST2 Tregs
CD8 THAP6+
CD4 Tn
CD4 Th1-22
CD4 TRM
CD4 Th17
CD8 TRM
CD4 Tregs
CD4 GZMK
NK/NKT
TCR
MAMU-I
OXPHOS
e
RTEs
Th2 Tregs
Tregs
CD4 Tn
Th17
ILC3s
CD8 Th-like
Th1-22
CD4 TRM
Th2
NK/NKT
CD8 TEMRA-MAIT
CD8 GZMK
CD4 GZMK
γδ-T cells
CD8 TRM
N-glycan biosynth.
Galactose metab.
Global GSL biosynth.
Pentose interconv.
OXPHOS
Tyr metab.
Phe metab.
Pyrimidine metab.
Ganglio GSL biosynth.
Glyoxylate metab.
TCA cycle
1C pool (folate)
1º bile acid biosynth.
Trp metab.
Nicotinamide metab.
Purine metab.
Glutathione metab.
Drug metab. enz.
CoA biosynth.
Pyruvate metab.
FA biosynth.
UFA biosynth.
FA deg.
Arg & Pro Metab.
Lacto GSL biosynth.
Cys Met metab.
Lys deg.
Glycerolipid metab.
KS biosynth.
Beta Ala metab.
Folate biosynth.
N metab.
Amino/Nuc sugar metab.
Porphyrin metab.
Retinol metab.
Gly Ser Thr metab.
Butanoate metab.
Ketone syn/deg
Val Leu Ile metab.
Ala Asp Glu metab.

Fig. S3

Supplementary Figure 3: Selected phenotypes of T cells and ILCs

(a) Expression of *TRAV* (n = 14), *TRBV* (n = 14), *TRDC* (n = 1) and *TRGV* (n = 2) transcripts amongst the T cells and ILCs. **(b)** Log_2 fold-change in expression of markers of T cell tissue residency. **(c)** NK and NKT-like cells are indicated by arrows alongside PRF1 and XCL1 expression within T cells and ILCs. The right-hand panel shows the sub-populations of the NK cell cluster, with top differentially expressed genes highlighted for *IFNG*$^+$, *IFNG*$^-$ NK cells and *ZBTB16*$^+$ NKT cells. **(d)** Log2 fold-change in expression of TCR complex (*CD247, CD3D, CD3E, CD3G*), MAMU Class I (*MAMU-A, MAMU-F*) and mitochondrially-encoded OXPHOS components (*COX1*, *COX2, COX3, COX4L1, COX5A, COX5B, ND2, ND5, ATP6*) amongst T cells and ILCs. **(e)** Relative expression of NanoString metabolic pathway genes by T cells and ILCs.

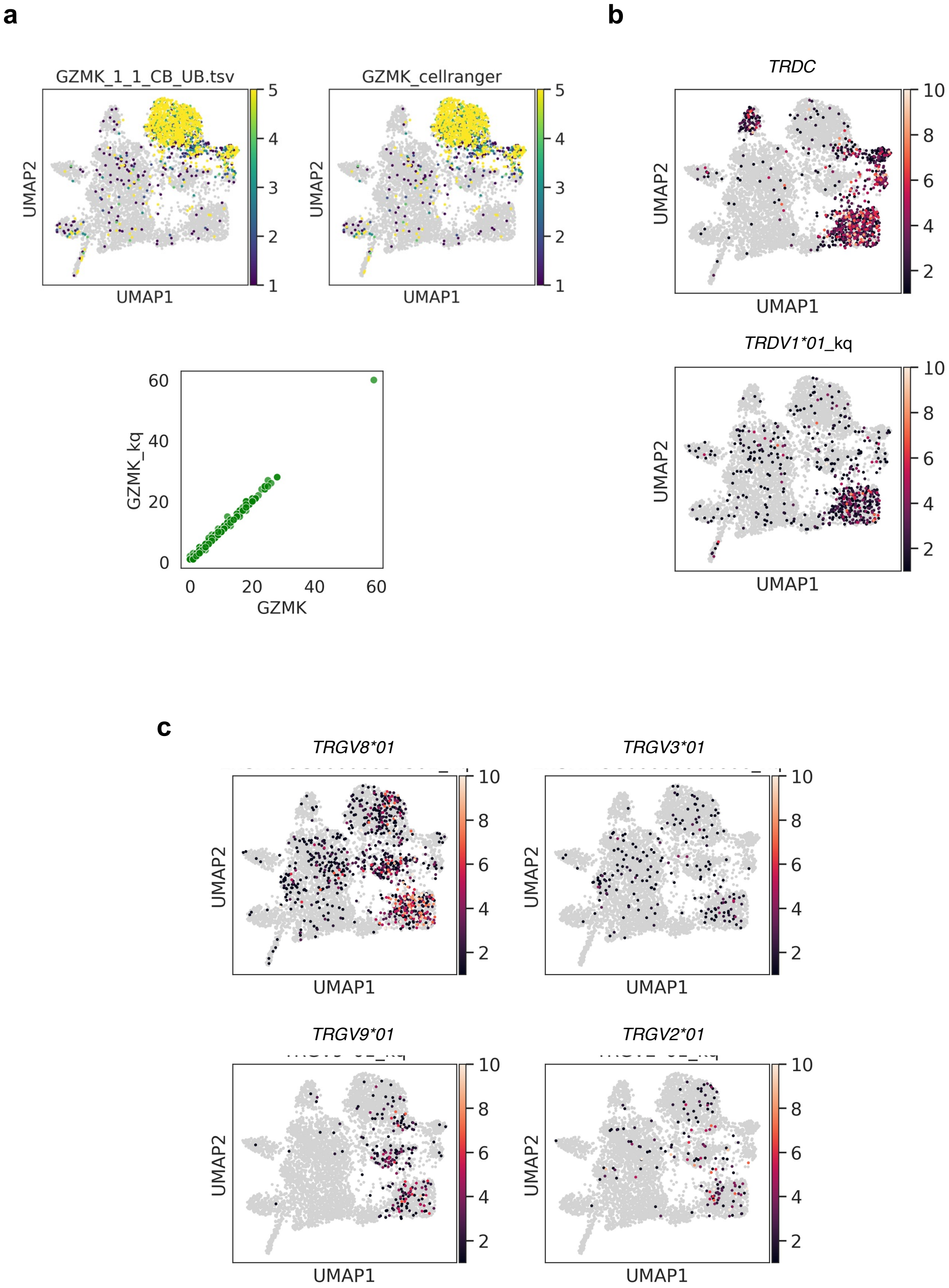
a
GZMK_1_1_CB_UB.tsv
GZMK_cellranger
UMAP1
UMAP2
GZMK_kq
GZMK
b
TRDC
TRDV1*01_kq
c
TRGV8*01
TRGV3*01
TRGV9*01
TRGV2*01

Fig. S4

Supplementary Figure 4: Building TRD and TRG consensus sequences from short reads

(a) Correlation between Kallisto (kq) and Cellranger (cr) outputs for the well-expressed *GZMK* transcript. **(b)** Kallisto projections of reads mapping to *TRDC* (ENSMMUG00000057791) and *TRDV1*01* (ENSMMUG00000054501). **(c)** Most-mapped genes in the *TRGV* database by Kallisto, illustrating a predominant role for *TRGV8*01*.

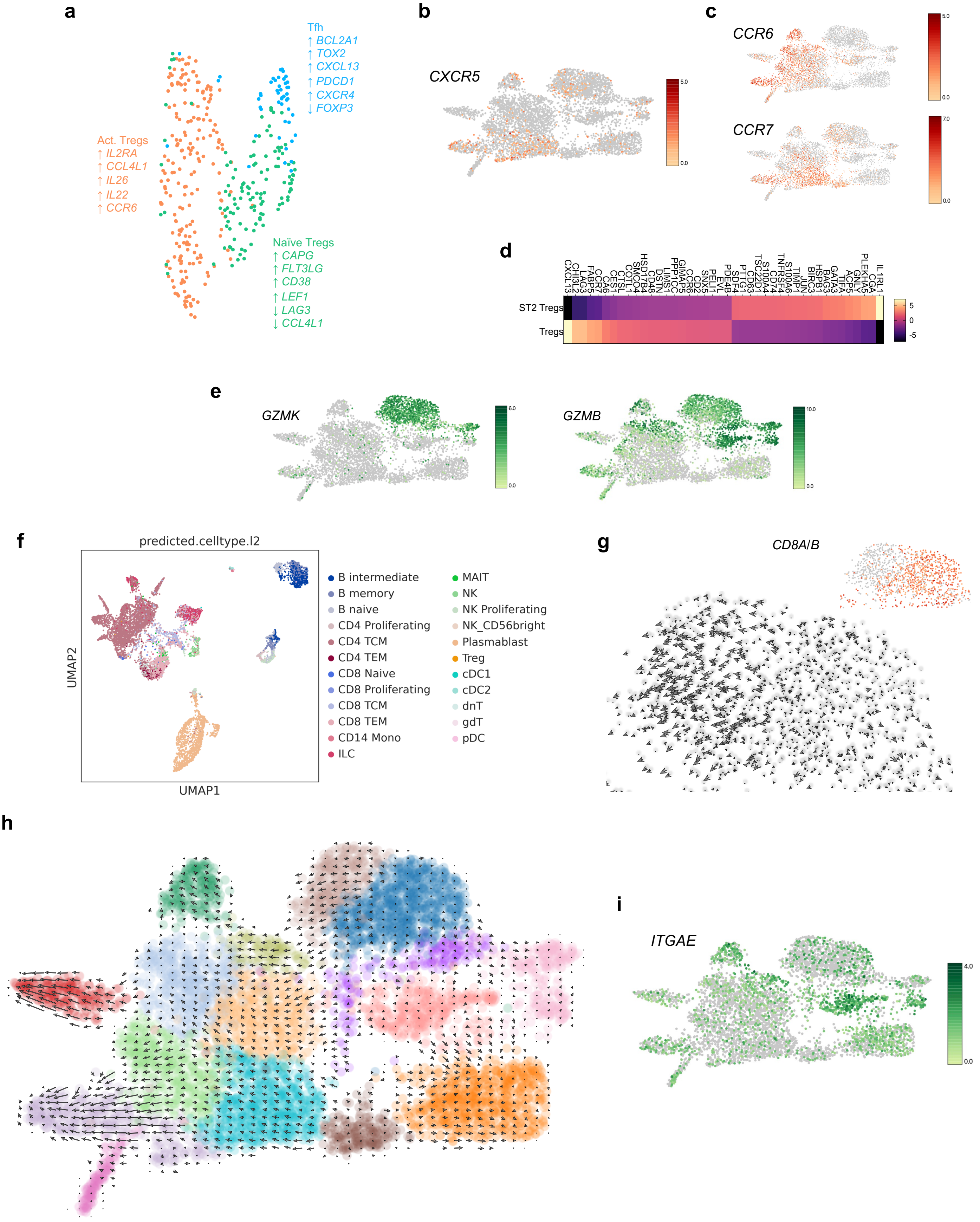

a
Tfh
↑ BCL2A1
↑ TOX2
↑ CXCL13
↑ PDCD1
↑ CXCR4
↓ FOXP3
Act. Tregs
↑ IL2RA
↑ CCL4L1
↑ IL26
↑ IL22
↑ CCR6
Naïve Tregs
↑ CAPG
↑ FLT3LG
↑ CD38
↑ LEF1
↓ LAG3
↓ CCL4L1
b
CXCR5
5.0
0.0
c
CCR6
5.0
0.0
CCR7
7.0
0.0
d
CXCL13 CHI3L2 LAG3 FABP5 CCR7 CA6 CES1 CTSL COTL1 SMCO4 HSD17B4 CD48 DSTN LIMS1 PPP1CC GIMAP5 CCR6 CD2 SNX5 PELI1 EVL PDE4B SDF4 PTTG1 CD63 TSC22D1 S100A4 CD74 TNFRSF4 S100A6 TIMP1 JUN BIRC3 HSPB1 BAG3 GATA3 TIFA ACP5 GNLY PLEKHA5 CGA IL1RL1
ST2 Tregs
Tregs
5
0
-5
e
GZMK
6.0
0.0
GZMB
10.0
0.0
f
predicted.celltype.l2
UMAP2
UMAP1
B intermediate
B memory
B naive
CD4 Proliferating
CD4 TCM
CD4 TEM
CD8 Naive
CD8 Proliferating
CD8 TCM
CD8 TEM
CD14 Mono
ILC
MAIT
NK
NK Proliferating
NK_CD56bright
Plasmablast
Treg
cDC1
cDC2
dnT
gdT
pDC
g
CD8A/B
h
i
ITGAE
4.0
0.0

Fig. S5

Supplementary Figure 5: Selected CD4$^+$ and CD8$^+$ T cell phenotypes in the cecum

(a) Treg sub-population re-clustering, showing key differentially expressed genes by Tregs and T_{fh} cells. **(b)** Expression of *CXCR5* by T cells and ILCs. **(c)** Expression of *CCR6* and *CCR7* by T cells and ILCs. **(d)** Heatmap of differentially expressed genes between Treg and ST2 Treg populations. **(e)** Expression of *GZMK*, *GZMB*, *CXCR4* and *CCR6* by T cells and ILCs. **(f)** Azimuth projection of a human PBMC reference on T cells and ILCs. **(g)** RNA velocity of *GZMK*$^+$ CD4$^+$ and CD8$^+$ T cells. *CD8A/B* expression across the two clusters is shown inset. **(h)** RNA velocities projected onto the T cell and ILC UMAP. **(i)** Expression of *ITGAE* by T cells and ILCs, showing highest expression within the CD8$^+$ TRM cluster.

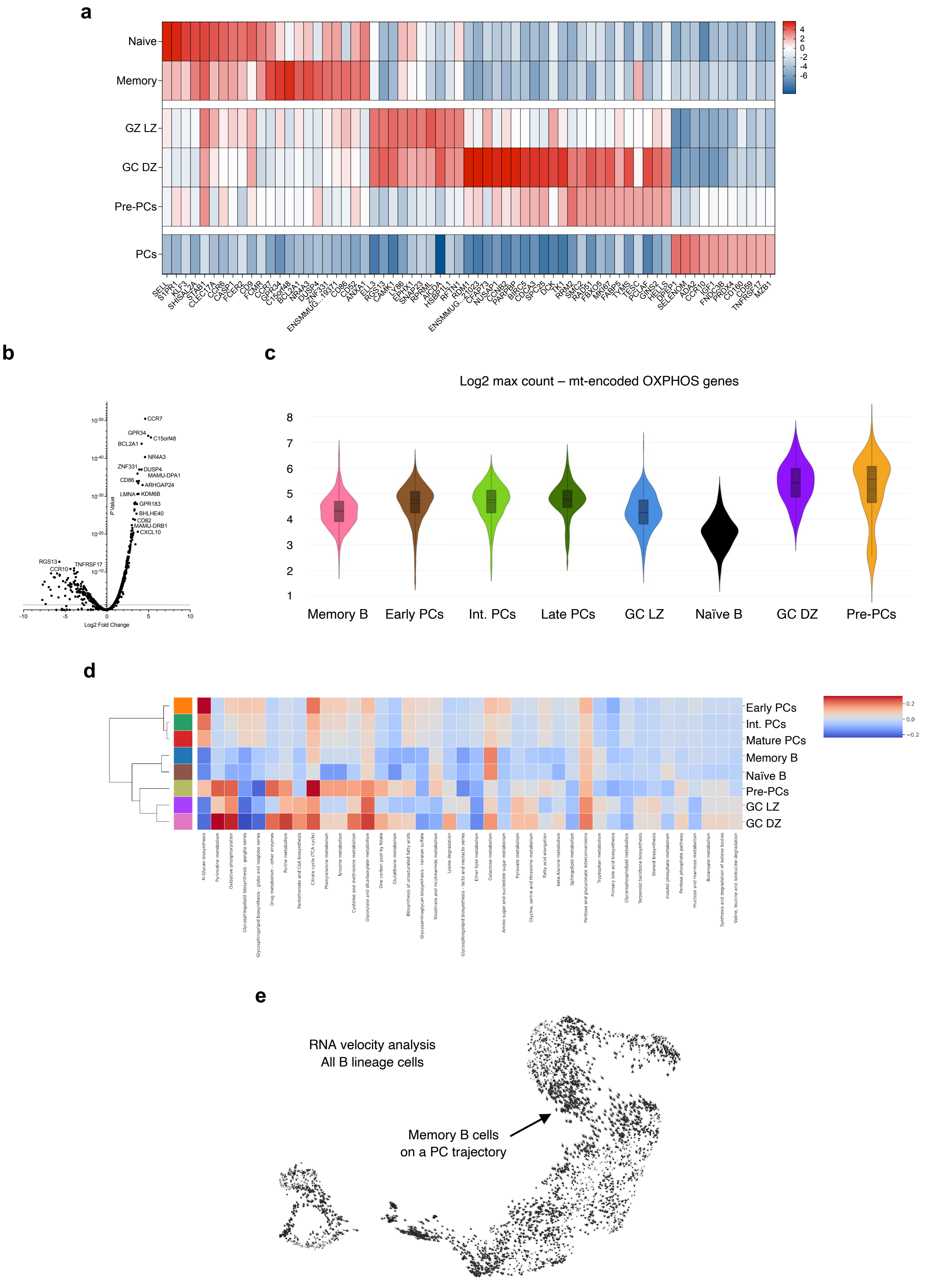
a
Naive
Memory
GZ LZ
GC DZ
Pre-PCs
PCs
4
2
0
-2
-4
-6
SELL
S1PR1
KLF3
SHISAL2A
STAB1
CLEC17A
CCR6
CASP1
FCER2
CD9
FCMR
CCR7
GPR34
C15orf48
BCL2A1
NR4A3
DUSP4
ZNF331
CD86
CD52
ANXA1
ELL3
RGS13
CAMK1
LY86
EPHX1
SNAP23
RPRML
AICDA
HSBP1L1
RFTN1
RDM1
CFAP73
NUSAP1
CCNB2
PARPBP
BIRC5
CDCA3
SPC25
DCK
TK1
RRM2
SMC2
RAD51
FBXO5
MKI67
FABP5
TYMS
TESC
PCLAF
GINS2
HELLS
DPEP1
SELENOM
ADA2
CCR10
IGF1
FNDC3B
PRDX4
CD160
CD59
TNFRSF17
MZB1
b
CCR7
GPR34
C15orf48
BCL2A1
NR4A3
ZNF331
DUSP4
MAMU-DPA1
CD86
ARHGAP24
LMNA
KDM6B
GPR183
BHLHE40
CD82
MAMU-DRB1
CXCL10
RGS13
TNFRSF17
CCR10
P Value
Log2 Fold Change
c
Log2 max count – mt-encoded OXPHOS genes
Memory B
Early PCs
Int. PCs
Late PCs
GC LZ
Naïve B
GC DZ
Pre-PCs
d
Early PCs
Int. PCs
Mature PCs
Memory B
Naïve B
Pre-PCs
GC LZ
GC DZ
e
RNA velocity analysis
All B lineage cells
Memory B cells
on a PC trajectory

Fig. S6

Supplementary Figure 6: B cell phenotypes in the rhesus macaque cecum

(a) Top differentially expressed genes by different B lineage clusters. **(b)** Genes differentially expressed by memory B cells, compared to all other B lineage cells. **(c)** Expression of mt-encoded OXPHOS genes by different B lineage clusters. **(d)** Relative expression of KEGG metabolic pathway genes by B lineage clusters. **(e)** RNA velocity analysis of B lineage cells.

a

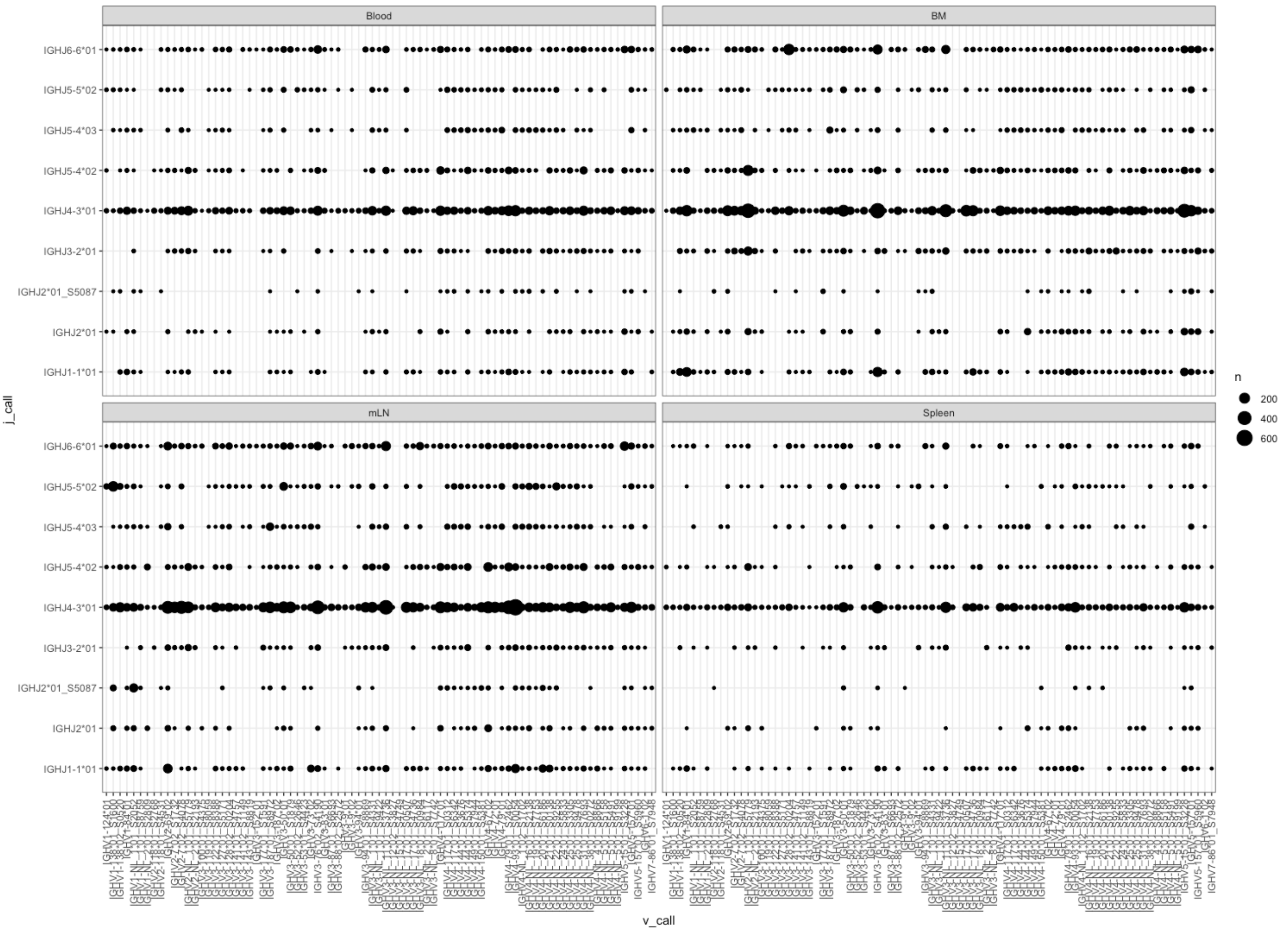

b

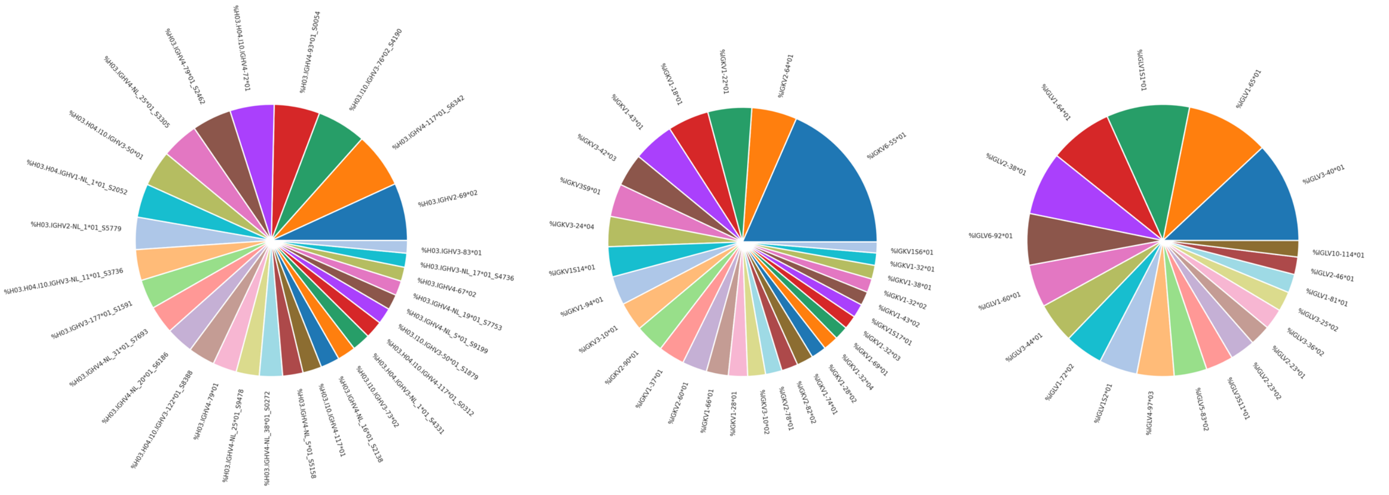

IGHV

IGKV

IGLV

Fig. S7

Supplementary Figure 7: Features of the cecal PC antibody repertoire

(a) V-J gene usage in different tissues of H03. RepSeq data IgA and IgG molecules after germline assignment using IgDiscover. **(b)** Heavy, kappa and lambda chain V gene usage by cecal PC antibodies.
